# A genetic profiling guideline to support diagnosis and clinical management of lymphomas

**DOI:** 10.1007/s12094-023-03307-1

**Published:** 2023-09-06

**Authors:** Margarita Sánchez-Beato, Miriam Méndez, María Guirado, Lucía Pedrosa, Silvia Sequero, Natalia Yanguas-Casás, Luis de la Cruz-Merino, Laura Gálvez, Marta Llanos, Juan Fernando García, Mariano Provencio

**Affiliations:** 1 Servicio de Oncología Médica, Grupo de Investigación en Linfomas, Hospital Universitario Puerta de Hierro-Majadahonda, IDIPHISA, Madrid, Spain; 2 Grupo Oncológico para el Tratamiento y Estudio de los Linfomas-GOTEL, Madrid, Spain; 3 Servicio de Oncología Médica, Hospital Universitario Puerta de Hierro-Majadahonda, IDIPHISA, Madrid, Spain; 4 Servicio de Oncología Médica, Hospital General Universitario de Elche, Alicante, Spain; 5 Servicio de Oncología Médica, Hospital Universitario San Cecilio, Granada, Spain; 6 Servicio de Oncología Médica, Facultad de Medicina, Hospital Universitario Virgen Macarena, Universidad de Sevilla, Instituto de Biomedicina de Sevilla (IBID)/CSIC, Seville, Spain; 7 Unidad de Gestión Clínica Intercentros de Oncología Médica, Hospitales Universitarios Regional y Virgen de la Victoria, Málaga, Spain; 8 Servicio de Oncología Médica, Hospital Universitario de Canarias, La Laguna, Sta. Cruz de Tenerife, Spain; 9 Servicio de Anatomía Patológica, Hospital MD Anderson Cancer Center, Madrid, Spain; 10 Servicio de Oncología Médica, Departamento de Medicina, Facultad de Medicina, Hospital Universitario Puerta de Hierro-Majadahonda, Universidad Autónoma de Madrid, IDIPHISA, Madrid, Spain

**Keywords:** Lymphoma, Next-generation sequencing, Diagnosis, Prognosis

## Abstract

The new lymphoma classifications (International Consensus Classification of Mature Lymphoid Neoplasms, and 5th World Health Organization Classification of Lymphoid Neoplasms) include genetics as an integral part of lymphoma diagnosis, allowing better lymphoma subclassification, patient risk stratification, and prediction of treatment response. Lymphomas are characterized by very few recurrent and disease-specific mutations, and most entities have a heterogenous genetic landscape with a long tail of recurrently mutated genes. Most of these occur at low frequencies, reflecting the clinical heterogeneity of lymphomas. Multiple studies have identified genetic markers that improve diagnostics and prognostication, and next-generation sequencing is becoming an essential tool in the clinical laboratory. This review provides a “next-generation sequencing” guide for lymphomas. It discusses the genetic alterations of the most frequent mature lymphoma entities with diagnostic, prognostic, and predictive potential and proposes targeted sequencing panels to detect mutations and copy-number alterations for B- and NK/T-cell lymphomas.

## Introduction

Mature lymphoid malignancies (Hodgkin (HL) and non-Hodgkin lymphomas (NHL)) are the most common hematological solid neoplasias. Thanks to the implementation of high-throughput molecular analysis, our knowledge of the molecular characteristics of lymphomas has been strengthened. Lymphoma classification is still mainly based on morphology, immunophenotype, and a few genetic characteristics. However, the new classifications of mature lymphoid neoplasms, the International Consensus Classification (ICC) of Mature Lymphoid Neoplasms [1] and the 5th World Health Organization (WHO) Classification of Lymphoid Neoplasms [2], have incorporated newly developed technologies to improve lymphoma classification and genetic alterations are now part of the criteria applied to lymphoma diagnosis.

Standardizing these high-throughput techniques to enable their full and successful clinical application is ongoing, but much controversy persists about the sequencing approach. One of the most critical issues is the method of choice and the composition of the sequencing panel. An ideal panel should be helpful in diagnosis, prognostication, therapy selection, and monitoring but small enough to be broadly and uniformly used. The ability to sequence the whole exome or genome is growing steadily. Nevertheless, a custom panel is currently the most accessible option to broaden the applicability of this approach. These targeted panels enable the analysis of a small number of genes in greater depth, with increased sensitivity and at a lower cost. Amplicon or capture-based sequencing panels could be used. The capture-based ones enable detection not only of single nucleotide variations (SNVs) and *indels* (insertions and deletions) but also of copy-number alterations (CNAs) and some structural variants. However, the detection of structural alterations needs further improvement. Additionally, standardization of sample management, panel composition, sequencing procedures, bioinformatic analysis, and variant interpretation is essential to produce a useful clinical tool.

This review aims to summarize the fundamental molecular characteristics of the various lymphoma types and to describe the genes relevant to each subtype that could be included in a massive sequencing or next-generation sequencing (NGS) panel.

## Mature B-cell lymphomas

### Chronic lymphocytic leukemia

Chronic lymphocytic leukemia (CLL) is a low-grade lymphoproliferative disorder characterized by the clonal proliferation and accumulation of mature, typically CD5 + B-cells within the blood, bone marrow, lymph nodes, and spleen [1–4].

More than 80% of CLL cases feature some cytogenetic abnormality and their detection by fluorescence in situ hybridization (FISH) stratify the patients into different risk groups [5]: deletion on the long arm of chromosome 13 (del(13q)), occurs in approximately 55% of cases [6, 7]; trisomy 12 is the second most frequent recurrent chromosomal aberration (10–20% of cases) [6, 8]; deletions on the long arm of chromosome 11 (del(11q)) are found in approximately 25% of chemotherapy-naïve patients with advanced disease and 10% of patients with early disease [9]; and deletion on the short arm of chromosome 17 (del(17p)) occurs in 5–8% of chemotherapy-naïve patients. Only the latter abnormality is considered a significantly negative prognostic factor [10–12]. Other frequent, recurrent abnormalities in CLL include 6q deletion (5%) and 2p gain (5–16%), among others [1, 13].

Somatic hypermutation (SHM) of the immunoglobulin heavy chain variable region (IGHV; < 98% similarity of the IGHV sequence, mutated CLL, M-CLL) confers a better prognosis than the absence of mutation (unmutated CLL, U-CLL) [5, 14, 15]. Recently, the mutation IGLV3-21^R110^ found in around 5–15% of CLL, can confer a poor prognosis, independently of the IGHV mutational status [16]. *TP53* mutations are found in 4–37% of CLL cases. They may occur alone or, more frequently, in combination with del(17p). They have been associated with chemo-refractoriness and reduced overall survival (OS) [17, 18]. NGS studies have helped identify mutations in other genes with prognostic relevance, such as *BIRC3*, *NOTCH1*, *SF3B1*, *MYD88*, *ATM*, *FBXW7*, *POT1*, *NFKBIE*, *CHD2*, *RPS15*, *IKZF3*, *ZNF292*, *ZMYM3*, *ARID1A*, and *PTPN11* [5, 19–22].

Richter transformation is defined as a transformation of CLL into aggressive lymphoma, most commonly diffuse large B-cell lymphoma (DLBCL). These patients typically have a poor response to traditional chemotherapy than de novo DLBCL. Moreover, their pattern of mutations is different from that of DLBLC-not otherwise specified (NOS). The risk of Richter transformation has been associated with prior therapy, U-CLL, *NOTCH1* mutations, del(17p) and del(11q) [23–25].

Mutations in *BTK*, *PLCG2*, and *CARD11* have been associated with resistance to BTK inhibitors [26, 27], whereas mutations in *BCL2* have been linked to venetoclax resistance [28]. Therefore, choosing between immunochemotherapy and targeted therapies for CLL heavily depends on 17p/*TP53* and IGHV status. Even though the treatment choice does not strictly require sequencing data, these data should be integrated into the decision and follow-up. As mentioned above, several genetic aberrations negatively impact prognosis, seem to confer a poorer response to conventional chemotherapy, and could be helpful when considering other treatment options based on BTK or BCL2 inhibition.

**Fig. 1:**
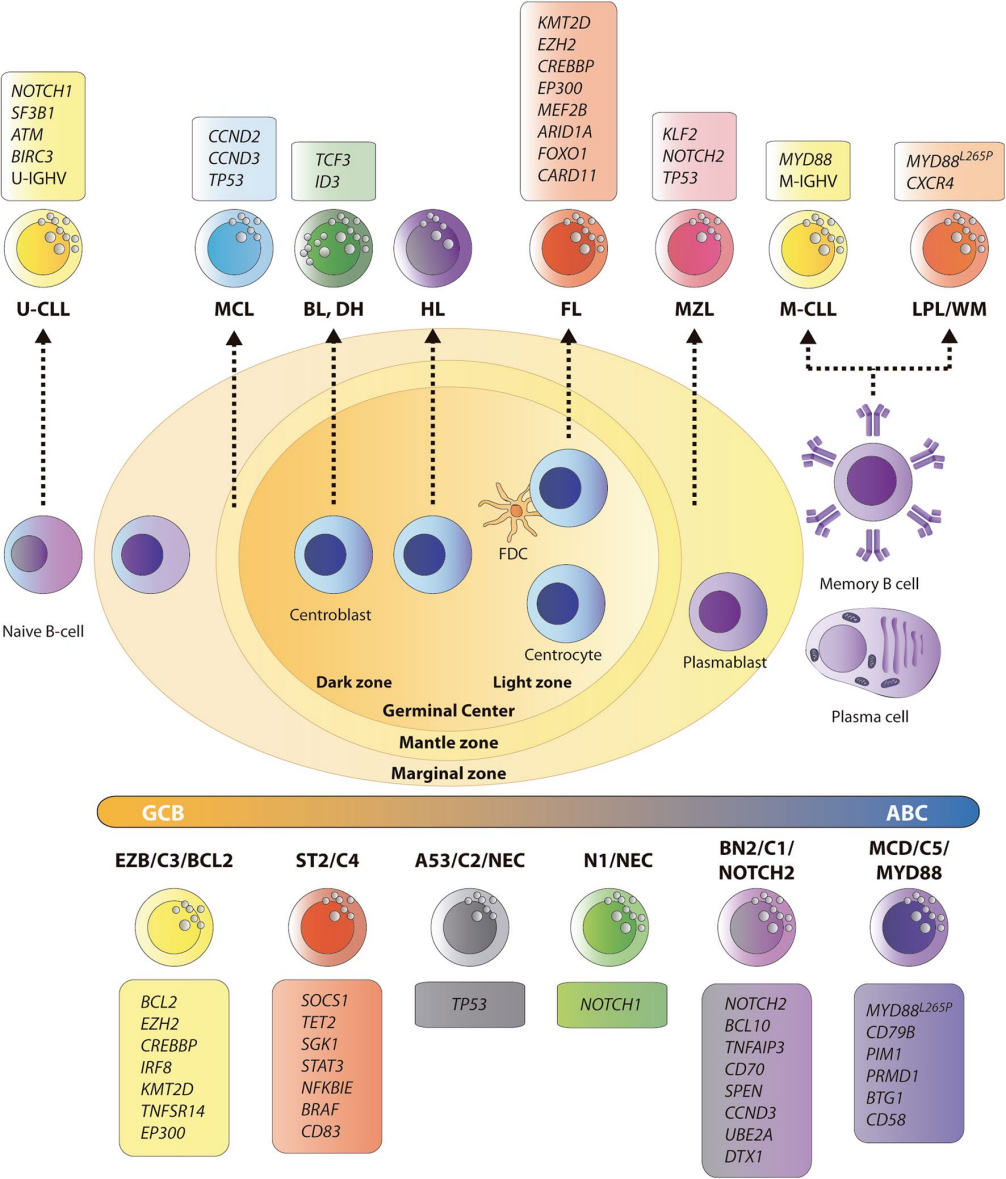
Mature B-cell malignancies: cell of origin and main genetic alterations. Schematic representation of B-cell maturation process throughout the germinal center from naïve B-cells to memory and plasma B-cells, and their derived mature B-cell lymphoma. The recently proposed genetic subtypes for DLBCL are represented in the lower part of the figure, with the genes defining each subtype and the relationship with the DLBCL COO classification. The most relevant mutated genes are indicated in the box associated with each subtype. *U*-unmutated; *M*-mutated; *CLL* chronic lymphocytic leukemia; *MCL* mantle cell lymphoma; *FL* follicular lymphoma; *MZL* marginal zone lymphoma; *DLBLC* diffuse large B cell lymphoma; *BL* Burkitt’s lymphoma; *DH* Double hit; *HL* Hodgkin lymphoma; *LPL/WM* lymphoplasmacytic lymphoma/Waldenström macroglobulinemia; *FDC* follicular dendritic cells; *COO* cell-of-origin; *GCB* germinal center B cell; *ABC* activated B cell

An ideal NGS panel for CLL should integrate the detection of IGHV SHM, gene mutations and CNAs (Table 1 and Fig. 1).

**Table 1:** Summary of genetic alterations in B-cell lymphomas and their clinical utility

B-cell Lymphoma		Genetic alterations	Clinical significance	Refs.
Chronic lymphocytic leukemia (CLL)		**IGVH SHM, Del(17p)/*TP53*, Del(11q),** Del(13q), trisomy 12**, *NOTCH1, SF3B1, ATM, BIRC3,**** NFKBIE, EGR2, MYD88, XPO1, CHD2*	DIAGNOSTIC/PROGNOSTIC	[5, 15, 17, 19, 22]
		***BTK, PLCG2, BCL2, TP53,**** CARD11*	PREDICTIVE	[26–28]
Lymphoplasmacytic lymphoma (LPL)		***MYD88***^**L265P**^***#, CXCR4***	DIAGNOSTIC/PROGNOSTIC/PREDICTIVE	[29, 32, 33]
Marginal zone lymphoma (MZL)	Spleenic MZL	***KLF2***, ***NOTCH2***, ***TP53***, *NOTCH1, MLL2, ARID1A, SIN3A, TNFAIP3, MYD88, CARD11*, **trisomies *3***, ***18***, Del(7q)	DIAGNOSTIC	[35, 37, 40]
	Nodal MZL	***KLF2, NOTCH2,**** KMT2D, PTPRP****,* trisomies* 3, 18***, *7*, *12*	DIAGNOSTIC	[38, 41]
	Extranodal MZL	**trisomies 3, 18, 12, T(11;18)#,** T(1;14), T(3;14), T(14;18) (IGH::MALT1*); TNFAIP3, CD79A, CD79B, CARD11, BIRC3, TRAF3, TNFRSF11A*	DIAGNOSTIC/PROGNOSTIC (m7-FLIPI)/THERAPY	[42–45]
Follicular lymphoma (FL)	FL	T(14;18); KMT2D, EZH2*#, CREBBP#, EP300#, MEF2B#, ARID1A#, FOXO1#, CARD11#	DIAGNOSIS/PROGNOSTIC^#^/THERAPY*	[54, 56]
		*NOTCH2, DTX1, UBE2A, HIST1H1E, MYC, TP53, CCND3, GNA13, S1PR2, P2RY8, POU2AF1, CDKN2A/B* loss	PROGNOSTIC (HT)	[58–61]
	Diffuse FL	***TNFRSF14, STAT6, CREBBP,**** EZH2*	DIAGNOSTIC	[47]
	Pediatric TFL	*TNFRSF14, MAP2K1*	DIAGNOSTIC	[50]
	Duodenal type FL	*TNFRSF14, CREBBP, EZH2*	DIAGNOSTIC	[49]
Mantle cell lymphoma (MCL)		**T(11;14), *CCND2* and* CCND3* rearr**	DIAGNOSTIC	[1, 2]
		**IGVH SHM, Del(17p)/*TP53,**** ATM, NOTCH1/2, KMT2D*	PROGNOSTIC	[64]
		**Del(17p)/*TP53****, BIRC3, TRAF2, NSD2, CARD11*	PREDICTIVE	[66–68]
Diffuse large B-cell lymphoma, not otherwise specified (DLBCL-NOS)		***NOTCH2, BCL10, TNFAIP3, UBE2A, CD70, CCND3, DTX1, BCL2, EZH2, CREBBP, TNFRSF14, KMT2D, IRF8, EP300, GNA13, MYD88, CD79B, PIM1, PIM2, PRDM1, BTG1, CD58, NOTCH1, SGK1, SOCS1, TET2, STAT3, TP53; MYC* rear*., BCL2* rearr*., BCL6* rear**	DIAGNOSTIC/PROGNOSTIC	[73–77]
Large B-cell lymphoma with IRF4 rearrangement		***IRF4* rear*,**** IRF4, CARD11, MYD88, CD79B*	DIAGNOSTIC	[69]
Large B-cell lymphoma with 11q aberration		**11q aberration,** *GNA13*	DIAGNOSTIC	[83]
High grade B-cell lymphoma (HGBCL)	HGBCL-DH-*BCL2*	***MYC* rear*., BCL2* rear*.,**** BCL2, KMT2D, CREBBP, TNFRS14, EZH2*	DIAGNOSTIC/PROGNOSTIC	[1, 2]
	HGBCL-DH-*BCL6*	***MYC* rear*., BCL6* rear**	DIAGNOSTIC	[1, 2]
	HGBCL-NOS	*MYD88, CD79B, TBL1XR1, TP53, KMT2D*	DIAGNOSTIC	[1, 2]
Burkitt Lymphoma (BL)	EBV +	***MYC rear.,**** MYC* aSHM*, CDKN2A, DDX3X*	DIAGNOSTIC	[100, 101]
		*TP53*	PROGNOSTIC	[102]
	EBV -	***MYC* rear*., TCF3, ID3,**** CDKN2A, DDX3X*	DIAGNOSTIC	[100, 101]
		*TP53*	PROGNOSTIC	[102]
Hodgkin Lymphoma (HL)		*XPO1, EP300, CREBBP, TP53, B2M, NFKBIE, TNFAIP3, STAT3, STAT6, PTPN1, ITPKB, GNA13, ARID1A, KTM2D, IGLL5, CSFR2B, BTK*	FUTURE	[108–110]

The most relevant alterations are indicated in bold

*Del* deletion; *SHM* somatic hypermutation; *EBV* Epstein–Barr virus; *DH* double hit; *NOS* not otherwise specified

HT: Histological transformation; rear.: rearrangement. Refs.: references. The most relevant alterations are indicated in bold

### Lymphoplasmacytic lymphoma/Waldenström macroglobulinemia

Lymphoplasmacytic lymphoma/Waldenström macroglobulinemia (LPL/WM) is an uncommon low-grade B-cell lymphoma, with an annual incidence of 3–4 cases per million people, characterized by bone marrow infiltration of clonal lymphoplasmacytic cells and the hypersecretion of immunoglobulin M [1, 2]. The lack of specific morphological, immunophenotypic, or chromosomal features requires the diagnosis to be made after excluding other small B-cell lymphomas.

Despite its rarity, our understanding of the biology of this disease has improved significantly in recent years with the identification of recurrent mutations in the *MYD88* and *CXCR4* genes [29]. More than 90% of LPL/MW cases bear the MYD88^L265P^ mutation. However, this is neither necessary nor specific for a diagnosis and can be seen in other B-cell lymphomas such as non-germinal center (GC) DLBCL, CLL, splenic marginal zone lymphomas, primary cutaneous DLBCL, leg type DLBCL, primary central nervous system DLBCL, or testicular DLBCL [30]. However, the co-occurrence of both mutations is highly suggestive of LPL/WM [31]. MYD88^L265P^ triggers tumor-cell growth through BTK, a target of ibrutinib [32]. *CXCR4* is mutated in 30% of patients with LPL/WM, is associated with shorter treatment-free survival, and confers resistance to ibrutinib [32–34]. Patients with *MYD88* and *CXCR4* mutations have important differences in disease presentation and response to therapy with ibrutinib: overall response rates are 100% for those with MYD88^L265P^ and without *CXCR4,* 85.7% for patients with both mutations and 71.4% for those without any mutation [32].

Other reported genetic alterations include deletion of 6q (40–60% of cases), mutations in *PRDM2* and *BTG1* (approximately 90% of cases), *HIVEP2*, *MKLN1*, *PLEKHG1*, *LYN*, *ARID1B*, *FOXP1*, and *ARID1A* [32]. Molecular mutational diagnostic approaches, especially NGS, have helped refine the diagnostic criteria for LPL/WM. Molecular testing can be used for risk stratification and treatment planning of LPL/WM, beyond diagnostic purposes (Table 1 and Fig. 1).

### Marginal zone lymphoma

Marginal zone lymphomas (MZLs) constitute a group of indolent B-cell lymphomas that arise from B lymphocytes in the marginal zone. They mainly compromise splenic MZL (SMZLs), with or without villous lymphocytes, nodal MZLs (NMZLs), and extranodal MZLs of mucosal-associated lymphoid tissue (EMZLs or MALTs). These are distinct clinical entities with specific diagnostic criteria and different genetic features, clinical behavior, and therapeutic implications [1, 2, 35]. MZLs account for approximately 10% of all NHLs derived from post-GC memory B-cells in the marginal zone of lymph nodes. The genomic landscape of MZL has been studied, revealing considerable overlap between mutated genes across distinct entities [1, 2, 36] (Table 1 and Fig. 1).

The most widely mutated genes in **SMZL** are *KLF2* and *NOTCH2*. Other frequent mutations affect *TP53* and *NOTCH1.* Other mutations leading to NF-κB pathway activation involving *TNFAIP3*, *CARD11*, *MYD88* (outside the p.265 hotspot), or *TRAF3* have been reported alongside alterations in chromatin remodelers such as *KMT2D*, *ARID1A*, and *SIN3A* [37, 38]. More than 30% of splenic MZLs are characterized by the deletion of 7q31–32 [36, 37, 39, 40].

**NMZL** and SMZL have several common alterations. Trisomies of chromosomes 3 and 18 co-occur in around 25% of SMZL and NMZL cases. The mutational profile of NMZL shows other recurrent clonal abnormalities like trisomies 7 and 12, and 6q deletion. Mutational analysis has identified mutations in *KLF2*, *PTPRD*, *KMT2D*, *NOTCH2*, *LRP1B*, *TET2*, and *TNFRSF14*. Other less frequent genetic alterations in NMZL include mutations in *BRAF*, *EZH2*, and *HIST1H1E* [38, 41].

The pathogenesis of **EMZL/MALT** is linked to several recurrent chromosomal aberrations, such as trisomies of 3, 12, and 18, which are found in 20–30% of cases. EMZL also presents recurrent chromosomal translocations. The most common is t(11;18)(p21;q21), which results in a functional chimeric fusion product between BIRC3 and MALT1 [36, 42]. It is associated with a low probability of response to antibiotics, *Helicobacter pylori*-negative, more advanced disease, and a lower risk of transforming to DLBCL. This translocation is specific for EMZL, since it has not been reported in SMZL or NMZL. Other common translocations are t(3;14)(p14;q32) (*FOXP1*::IGH), which is found in around 10% of EMZL cases; t(14;18)(q32;q21) (IGH::*MALT1*), which is present in 15–20% non-gastrointestinal EMZLs; and t(1;14)(p22;q32) (*BCL10*::IGH), which is a rare translocation found in 1–2% of EMZLs [1, 2, 43]. Homozygous deletion of the chromosomal band 6q23, involving *TNFAIP3* (A20), has been described in EMZL and may contribute to lymphomagenesis by inducing constitutive NF-κB activation [44]. *MYD88* mutation is detected in ocular adnexal MALT lymphoma (5% of cases) and can activate NF-κB, STAT3, and AP1 transcription factors [43]. Apart from the mutations of *TNFAIP3* and *MYD88*, other alterations in NF-κB regulators have been identified (*CD79A*, *CD79B*, *CARD11*, *BIRC3*, *TRAF3*, and *TNFRSF11A*) [43]. The result of the recurrent genetic alterations mentioned above in the activation of the NF-κB activation pathway represents a possible therapeutic target for MALT lymphomas [45].

### Follicular lymphoma

Follicular lymphoma (FL) is a malignancy derived from GC B-cells and the most common indolent B-cell lymphoma. It remains an incurable malignancy, but OS may last 20 years [46]. A key hallmark of FL is the t(14;18)(q32;q21) IGH::*BCL2* translocation, the first hit in its oncogenesis. Recently, the new WHO and ICC classifications, recognized unique FL entities, such as in situ follicular B-cell neoplasm, duodenal-type FL, primary cutaneous follicle cell lymphoma, pediatric-type FL, and testicular FL.

A distinctive diffuse follicular lymphoma (dFL) variant lacking t(14;18) was first described in 2009 [47]. In a recent study, NGS analysis identified two molecular clusters: one was characterized by *TNFRSF14* mutations, and the other showed few genetic alterations, a subgroup with *STAT6* mutations concurrent with *CREBBP* mutations without *TNFRSF14* and *EZH2* mutations [48]. These findings suggest dFL might represent a subtype of t(14;18)-negative FL.

Gastrointestinal FL, especially duodenal-type FL (DTFL), frequently occurs as extranodal FL. This lymphoma is commonly found in the second part of the duodenum and exhibits indolent clinical behavior. It is morphologically and immunophenotypically indistinguishable from typical FL. More widespread use of NGS has identified that the mutation frequencies of recurrently mutated genes, including *TNFRSF14*, *CREBBP*, and *EZH2,* were not significantly different from typical FL, but *KMT2D* was less commonly mutated in DTFL [49].

Pediatric-type FL (PTFL) occurs in younger patients and shows a preference for the head and neck. Some studies have used NGS technologies to describe a specific mutational profile in PTFL distinct from those of other lymphomas, including typical FL. *TNFRSF14* and *MAP2K1* are the genes most frequently reported to be mutated in PTFL. One or the other is present in about 80% of cases, but they do not usually co-occur. This finding indicates that both genes are essential for the pathogenesis of PTFL [50].

In 2011, Morin et al. described frequent mutations of *KMT2D* and other chromatin-modifying genes (CMGs)[51]. These genes code for histone methyltransferases (EZH2, KMT2D) or histone acetylases (MEF2B, CREBBP, EP300) [30]. Alterations in these genes have been established as a central genetic hallmark of FL and are critical for determining GC and post-GC B-cell fate. The characteristic distinguishing FL from other B-cell lymphomas is the high rate of mutations in CMGs. The *KMT2D* gene is the most recurrently mutated CMG in FL (72%), followed by *CREBBP* (∼65% of FLs) and *EP300* (15%) [52, 53]. Mutations of *EZH2* are found in 25% of FLs; it has prognostic relevance and is currently investigated as a druggable target of therapeutic potential. EZH2 is a regulator of the GC phenotype, and mutations in this gene block B-cells and stop their differentiation into plasma cells.

Therapeutic targeting of epigenetic deregulation is an attractive concept. However, the most common CMG mutations (*KMT2D* and *CREBBP*) are loss of function/loss of protein events, which are difficult to target with drugs. This constraint has led researchers to focus on *EZH2* mutations, with various companies developing inhibitors for EZH2 [54]. In a recent phase II study, tazemetostat, an oral inhibitor of EZH2, showed anti-tumor activity in patients with relapsed or refractory FL. Patients with or without mutations in *EZH2*, received tazemetostat and objective responses were observed in 69% of patients with mutated *EZH2*, and 35% of patients with wild-type *EZH2* [55].

The Follicular Lymphoma International Prognostic Index (FLIPI) is the most widely used risk predictor. For failure-free survival, Pastore et al. proposed a clinicogenetic risk model, the m7-FLIPI score, which included the mutational status of seven genes (*ARID1A, EZH2, EP300, FOXO1, MEF2B, CREBBP* and *CARD11*), the Eastern Cooperative Oncology Group (ECOG) performance status and FLIPI [56]. m7-FLIPI defined a high-risk group with a 5-year failure-free survival rate of 38%, compared with 77% for the low-risk group, in patients who received first-line treatment with a combination of rituximab and chemotherapy (CVP or CHOP). But low-risk m7-FLIPI does not indicate a more indolent disease course, as all patients required chemotherapy. However, several studies concluded that the prognostic value of the m7-FLIPI clinicogenetic model seems to be dependent on the therapeutic regimen [57] (Table 1 and Fig. 1).

Histological transformation (HT) is reported to occur in 15–30% of patients with FL. HT refers to the evolution of an FL to a clinically aggressive lymphoma, such as DLBCL or Burkitt lymphoma, which is usually associated with poor prognosis and chemotherapy resistance. HT has been associated with alterations deregulating cell-cycle progression and DNA-damage responses (*CDKN2A*/*B*, *MYC*, and *TP53*) [58, 59], or other genes more commonly mutated in transformed samples than in FL tumors (*CCND3*, *GNA13*, *S1PR2*, and *P2RY8*) [60]. A study of FL samples from patients who did or did not transform discovered that the presence of mutations in four genes (*NOTCH2*, *DTX1*, *UBE2A,* and *HIST1H1E*) [61] was associated with a shorter time to transformation when mutated in the FL biopsies at diagnosis. This study also identified mutated genes enriched at transformation, like *POU2AF1*, which has roles in GC architecture and migration [61].

### Mantle cell lymphoma

Mantle cell lymphoma (MCL) accounts for about 6% of NHL cases. The disease has two clinical presentations: common conventional MCL (cMCL) (90% of patients), which usually has an aggressive clinical course (SOX-11-positive cells and an unmutated IGHV), and an indolent clinical presentation (10% of patients), which generally presents as non-nodal leukemic phase (nnMCL) (SOX-11-negative, mutations of *CCND1* and *TLR2*, and somatic hypermutation of IGHV) (2,3). MCL is typically an aggressive and incurable B-cell malignancy, but some patients may follow an indolent clinical course.

MCL is characterized by the t(11;14)(q13;q32) translocation, leading to the overexpression of Cyclin D1 (CCND1), which is detected in nearly 95% of cases. Although the detection of CCND1 helps support an MCL diagnosis in unclear mature B-cell neoplasms, FISH is the current gold standard assay used to identify recurrent cytogenetic alterations, although this methodology may not detect complex or cryptic rearrangements [62, 63]. The few cases without this characteristic *CCDN1* rearrangement are characterized by *CCND2* or *CCND3* translocations [1].

Some studies have identified significantly mutated genes such as *ATM*, and the tumor suppressor *TP53*, but also found *NOTCH2* mutations in aggressive tumors with a worse prognosis [64].

MIPI (MCL International Prognostic Index) is based on a weighted sum of performance status, age, lactate dehydrogenase (LDH) levels and white blood cell count. Additional modifications, such as “MIPI genetic” (MIPIg), are being explored to refine this score. MIPIg is associated with an increased risk of progression and death when mutations of *KMT2D* and deletion or mutation of *TP53* are present [65]. At diagnosis, the frequency of *TP53* mutations is about 11–25% but increases to 45% at relapse. *TP53* deletion (determined by FISH) and *TP53* mutations were associated with the worst survival [66] (Table 1 and Fig. 1).

Ibrutinib-refractory MCL patients exhibit poor survival and lack an optimal management strategy. Some studies have investigated the relationship between mutations in *BIRC3*, *TRAF2*, and *CARD11* genes, MCL progression, and ibrutinib resistance [67]. Recently, a study explored the mutational profile in a subset of patients who developed disease progression or disease transformation on ibrutinib treatment. Using targeted NGS, they detected *TP53* alterations in 75% of patients after progression on ibrutinib. They found mutations in chromatin-modifier genes, such as *NSD2* in 75% of patients with transformed MCL on ibrutinib therapy. They concluded that *NSD2* mutations are involved in altered methylation and chromatin dysfunction, leading to aberrant gene expression with pathological significance in MCL progression and ibrutinib resistance [68].

### Diffuse large B-cell lymphoma

DLBCL is the most common subtype of NHL (30–35% of cases), characterized by large mature B-cell morphology and phenotype [1, 2]. DLBCL is heterogeneous in its clinical behavior and pathological and molecular diagnosis. The most common type of DLBCL (80%) is DLBCL- NOS, which has no specific clinical presentation or pathology. Three molecular subtypes have been recognized by the WHO classification based on the cell of origin (COO): germinal center (GCB), activated B-cell (ABC) or non-GCB, and unclassifiable DLBCL [1, 69]. This classification has a prognostic value, with ABC-DLBCL associated with poorer outcomes [70]. However, the COO does not imply different treatments, R-CHOP (rituximab, cyclophosphamide, adriamycin, vincristine and prednisone) being the backbone of therapy for all subtypes [71]. Up to 40% of DLBCLs will be refractory to first-line treatment or will recur [72].

Several researchers have recently proposed new genetic groups with broad concordance, suggesting that mutational analysis could be a promising alternative to classify DLCBL with prognostic and theragnostic values [1, 2, 73–76]. Each genetic cluster harbors a distinct mutational profile. Schmitz et al*.* defined the following subtypes: MCD (co-occurrence of *MYD88*^L265P^ and *CD79B* mutations), BN2 (with *BCL6* fusions and *NOTCH2* mutations), N1 (*NOTCH1* mutations), EZB (*EZH2* mutations and *BCL2* translocations), A53 (*TP53* mutations and deletions) and ST2 (*SGK1* and *TET2* mutations) [73, 74]. In parallel, another approach distinguished five subsets of DLBCL, including two ABC-DLBCL groups, one with low risk and a possible marginal zone origin (C1), and the other a high-risk group (C5) enriched in cases with mutations in *MYD88*, *CD79B*, and *PIM1*; two subsets of GC-DLBCLs with favorable (C4) and poor (C3) outcomes, and an ABC/GC-independent group (C2) with biallelic inactivation of *TP53*, *CDKN2A* loss, and associated genomic instability [75]. To unify both classifiers, Lacy et al. established the subtypes NOTCH2, MYD88, BCL2, TET2/SGK1, and SOCS1/SGK1, according to the mutated genes that are most highly enriched in each one [76]. However, all these studies are mainly based on whole-genome sequencing and are not applicable in routine clinical practice. Hence, some efforts have been made to propose easy and feasible classifiers built with a combination of mutational and translocation data of a selected set of genes representing each genetic subtype [77, 78].

A consensus may soon be reached to develop an NGS panel for assigning genetic groups (Tables 1 and 2, and Fig. 1).). To move towards personalized medicine, it should include at least the analysis of the *MYC*, *BCL2*, and *BCL6* rearrangements, COO determination at diagnosis, and the chosen NGS panel. Although there is no agreement about the classification of the genetic subtypes, an adequate preliminary NGS panel could be designed, including a set of common genes from the genetic classifiers: *MYD88*, *CD79B*, *PIM1*, *PIM2*, *PRDM1*, *BTG1*, *CD58*, *ETV6*, and *TBL1XR1* for the MCD subtype; *NOTCH2*, *TNFAIP3*, *BCL10*, UBE2A, *CD70*, *CCND3*, and *DTX1* for the BN2 subtype; *EZH2*, *CREBBP*, *TNFRSF14*, *KMT2D*, *BCL2*, *IRF8*, and *EP300* for the EZB subtype; *SGK1*, *SOCS1*, *TET2*, *NFKBIA*, and *STAT3* for the ST2 subtype, and *NOTCH1* for the N1 subtype [77, 78].

**Table 2:** DLBCL genetic subtypes

Genetic types	Subtypes	Main alterations	COO	Clinical outcome [73–77]
MCD/C5/MYD88		*MYD88*^L265P^*, CD79B, PIM1, PRMD1, BTG1. CD58*	ABC	Bad prognosis
BN2/C1/NOTCH2		*BCL6* rear.*, NOTCH2*, *BCL10, TNFAIP3, CD70, SPEN, CCND3, UBE2A, DTX1*	ABC / GCB	Intermediate prognosis
EZB/C3/BCL2	EZB	*BCL2* rear., *BCL2*, *EZH2*, *CREBBP*, *IRF8*, *KMT2D, TNFSR14, EP300. MYC* rear	GCB	Good prognosis
	EZB-MYC		DH	Bad prognosis
N1/NEC		*NOTCH1*	Mostly ABC	Bad prognosis
A53/C2/NEC		*TP53,* aneuploidy	Mostly ABC	Intermediate prognosis
ST2/C4		*SOCS1, TET2 & SGK1, STAT3, NFKBIE, BRAF, CD83*	Mostly GCB	Good prognosis

Integration of the genetic subtypes described by Wright [74], Chapuy [75], and Lacy [76] and their relation to the cell-or-origin and patient clinical outcome

*COO* cell-of-origin; *ABC* activated B-cell; *GCB* germinal center B-cell; *DH* double hit; *rear*. rearrangement

Extranodal DLBCL cases arising in immune-privileged sites, such as primary DLBCL of the central nervous system (PCNSL), primary DLBCL of the testis, primary cutaneous DLBCL, leg type, and other related entities, such as breast DLBCL [79], have similar molecular features and there are some recent controversies about their classification [1, 2]. Most of the lymphomas in these locations are non-GCB/ABC type and seem to share common molecular features such as the high prevalence of MYD88^L265P^ and *CD79B* mutations that characterize the DLBCL-MCD/C5/MYD88 genetic subtype.

DLBCL associated with viral agents (EBV-associated, HHV-8-associated) are rare and, in most instances, their diagnosis is based on the clinical presentation and pathological features; a set of mutated genes possibly specific for this disease has been described in EBV-positive DLBCL, including *CCR6*, *CCR7*, *DAPK1*, *TNFRSF21*, *CSNK2B* and *YY1* [80], as well as common *PD-L1* genetic aberrations [81]

Large B-cell lymphoma (LBCL) with the *IRF4* rearrangement occurs most commonly in younger patients [1, 2]. It prefers the lymphoid tissue of Waldeyer’s ring and head and neck lymph nodes [82]. In a study with pediatric LBCL, the subtype with *IRF4* rearrangement had a GCB phenotype with frequent mutations in *IRF4* and NF-κB pathway genes (*CARD11*, *MYD88*, *CD79B*) [69].

LBCL with 11q aberration [1] (named “HGBCL with 11q aberrations” in the 5th WHO classification) [2] was classified as Burkitt-like lymphoma with 11q aberration in the previous WHO classification. The mutational pattern is more like that of GCB-DLBCL than Burkitt-like, lacks *MYC* rearrangements, and shows frequent mutations in *GNA13.* Genomic alterations affecting the ID3-TCF3 complex are quite exceptional in LBCL-11q [83].

### High-grade B-cell lymphoma

The recently revised 5th edition of the WHO Classification of Hematolymphoid Tumors and the ICC now recognize the following groups: high-grade B-cell lymphoma (HGBCL) with *MYC* and *BCL2* rearrangements (with or without *BCL6* rearrangement, HGBCL-DH-*BCL2*) [1, 2], a provisional entity with *MYC* and *BCL6* rearrangements (HGBCL-DH-*BCL6*) [1] and HGBCL-NOS.

The mutational findings in the HGBCL-DH-*BCL2* lymphoma subtype are relatively homogeneous and similar to those of FL, harboring frequent molecular abnormalities in *BCL2*, *KMT2D*, *CREBBP*, *TNFRS14*, and *EZH2*. Several recent studies described DLBCL cases with a gene expression signature (GEP) similar to that of HGBCL (DH-like GEP signature) [84–86] and have frequent mutations in *MYC*, *BCL2*, *DDX3X*, *TP53*, and *KMT2D* [85] (Table 1 and Fig. 1).

Lymphoid neoplasms with dual *MYC* and *BCL6* rearrangements are now considered a subtype of DLBCLNOS, or HGBCL, and their gene expression profiles are highly heterogeneous and unrelated to those of DLBCL/HGB-cell lymphomas with *MYC* and *BCL2* rearrangements.

HGBCL-NOS is recognized as another subtype and covers cases that cannot be included in the other entities. From a molecular perspective, it is a heterogeneous category that includes activated B-cell lymphomas with mutations of *MYD88*, *CD79B*, or *TBL1XR1*. The most frequent mutations are found in *TP53* and *KMT2D*. Gene-expression profiling showed that approximately half the cases of HGBCL-NOS harbor the previously described DH-like GEP signature [85–87].

### Burkitt’s lymphoma

Burkitt’s lymphoma (BL) is a highly aggressive mature B-cell NHL characterized by rapid proliferation [1, 2, 88]. It accounts for 1–2% of adult lymphomas, whereas it is a common pediatric neoplasm [89].

Historically, three BL clinical variants have been described and are currently recognized by the WHO classification: endemic, non-endemic or sporadic, and immunodeficiency associated. The three subtypes present identical morphological and immunophenotypical features but are clinically and epidemiologically different [1, 2, 90]. All clinical variants share pathological features. A defining feature of BL is the constitutive overexpression of MYC due to a translocation of this oncogene along with one of the three immunoglobulin genes located on chromosomes 14, 2 and 22, accounting for nearly 80%, 15%, and 5% of cases, respectively. Other typical lymphoma translocations, such as *BCL2* and *BCL6*, do not occur in this entity [2, 88]. However, MYC deregulation is not sufficient for lymphomagenesis. Therefore, other genetic alterations are commonly detected by next-generation techniques [91, 92].

Distinguishing BL from other HGBCLs is an important challenge in clinical practice that must be addressed. Gene expression profile studies have revealed a distinct BL signature, making it an independent entity [93–95]. Examining the BL genetic landscape, several studies have demonstrated that ID3/TCF3-dependent centroblast gene expression program, tonic PI3K-AKT-mTOR signaling, and cell cycle deregulation and apoptosis are essential mechanisms for BL lymphomagenesis. Inactivating mutations in tumor-suppressor genes, such as *TP53*, *CDKN2A*, and *DDX3X*, are recurrent in BL [92, 96, 97]. Moreover, TP53 inactivation has been reported as a potential prognostic biomarker because these mutations were enriched in refractory patients [96]. Likewise, the higher prevalence of BL among males may be partially accounted for by inactivating alterations in *DDX3X*. Loss-of-function *DDX3X* mutations moderate MYC-driven global protein synthesis, and established malignant cells restore full protein synthetic capacity by aberrant expression of their Y chromosome homolog (DDX3Y), whose expression is usually restricted to the testis [97]. Cell migration and dissemination disturbances are partly due to the inactivation of P2RY8 and GNA13 [98], and high levels of proliferation are due not only to CCND3 activation but also to CDKN2A inactivation [92]. Another oncogenic mechanism is the constitutive activation of BCR signaling by mutations in the transcription factor *TCF3* and its negative regulator *ID3*.

Consequently, BCR signaling activates PI3K-AKT-mTOR signaling, highlighting alterations targeting *PTEN* and *FOXO1*, essential for BL survival [92, 99]. Finally, although various chromatin regulators are recurrently mutated in BL, mutations in *EZH2*, *CREBBP*, and *KMT2D* are rarely observed, unlike in GCB-DLBCL [100]. Furthermore, studies searching for new BL subdivision strategies with clinical implications suggest that EBV status could be an up-and-coming approach [100, 101]. EBV-positive BL shows significantly higher levels of aberrant SHM, fewer driver mutations, particularly in the apoptosis pathway, and fewer mutations in *TCF3* and *ID3* [2, 100] (Table 1 and Fig. 1).

In fact, the new WHO classification recommends that EBV-positive and EBV-negative BL be recognized as distinct entities [2]. Regarding differential diagnosis, identification of *MYC* translocations for BL diagnosis and the absence of the typical chromosome 11q-gain/loss pattern observed in Burkitt-like lymphoma are mandatory. As for NGS, it would be recommendable to study differential mutational patterns in *TCF3* and *ID3*, which are recurrently mutated in BL, and *EZH2*, *CREBBP*, and *KMT2D,* which are mutated in other lymphomas. Moreover, *TP53* mutational status could provide prognostic information [102].

## Hodgkin lymphoma

Hodgkin lymphoma (HL) is a clonal neoplasm derived from B-cells in most cases. HL constitutes 25–30% of all lymphomas and is subdivided into classic Hodgkin lymphoma (cHL) (95% of cases) and nodular lymphocyte-predominant Hodgkin lymphoma (NLPHL) (5% of cases). Unlike other tumors, neoplastic cells (Hodgkin and Reed–Sternberg [HRS] and lymphocytic and histiocytic [L&H] cells, in the case of NLHPLN) account for less than 1–2% of the total cellularity. The ICC and new WHO classifications propose “nodular lymphocyte predominant B-cell lymphoma” (NLPBL) as a new term for NLPHL [1, 2], based on the significant biological and clinical differences from cHL and its close relationship to T-cell/histiocyte-rich large B-cell lymphoma [103].

cHL, primary mediastinal large B-cell lymphoma, and mediastinal gray-zone lymphomas are related diseases with common genetic alterations, phenotypes, and clinical features, including anterior mediastinal involvement [1]. cHL is a monoclonal proliferation of HRS cells (and their variants) accompanied by a reactive microenvironment, the recognition of both elements being essential for its diagnosis. Cytogenetically, besides aneuploidy and hyper-tetraploidy, HRS cells show recurrent chromosomal imbalances, including gains of 2p13 (*REL*), 9p24.1 (*CD274* (PDL1), *PDCD1LG2* (PDL2), JAK2), 17q21 (*MAP3K14*), and loss of 6q23-q24 (*TNFAIP3*) [104–107].

The scarcity of tumor cells has hampered the genetic characterization of cHL. However, genetic studies based on tissue microdissection or cell isolation by flow cytometry have shown the dysregulation of specific pathways rather than mutations in specific genes. Studies have revealed recurrent somatic mutations in NF-κB pathway components (*TNFAIP3, NFKBIA, NFKBIA, REL*); the JAK/STAT pathway (*SOCS1, PTPN1, STAT6, STAT3, CSF2RB*) [108–111]; epigenetic regulators such as *EP300*, *CREBBP* [108], and *TP53;* and regulators of immune escape such as inactivating mutations in the gene of the MHC class 1 component B2M, and the MHC class 2 transactivator (C2TA) [110, 112], and the *FAS* gene [113], which favor the evasion of apoptosis and cell proliferation. Other relevant signaling pathways in cHL pathogenesis include MAPK/ERK, AP1, PI3K/AKT, and NOTCH [114] (Table 1 and Fig. 1).

## Mature T-cell and NK-cell lymphomas

**Fig. 2:**
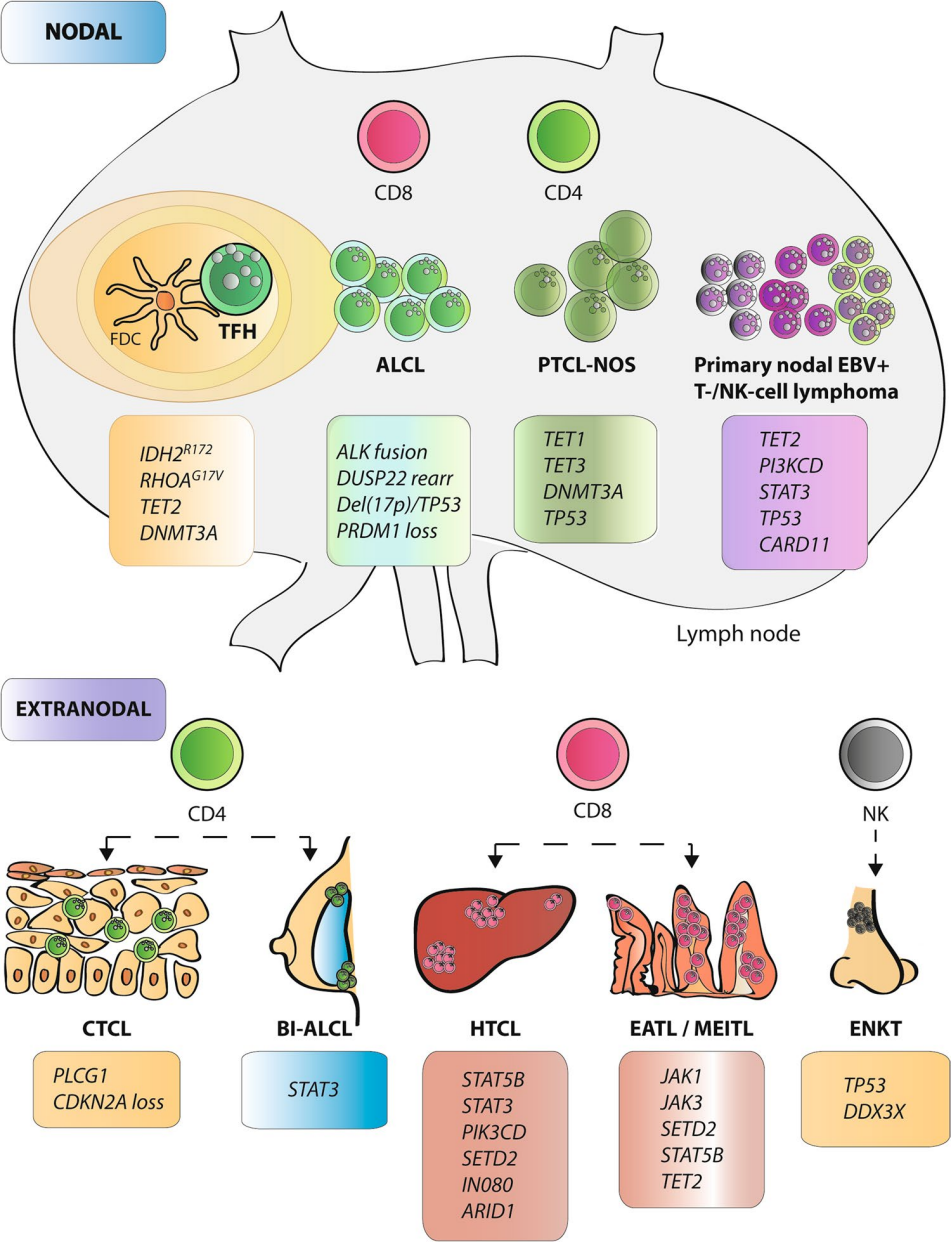
Essential genetic alterations in T/NK-cell malignancies. Schematic representation of T-cell and NK-cell derived malignancies, including the lymphocyte subtype they are derived from, and their pathological location in nodal or extranodal sites. The main genetic alterations of each entity are indicated in the box associated with each subtype. *TFH* follicular helper T-cell; *FDC* follicular dendritic cells; *AITL* angioimmunoblastic lymphoma; *NOS* not otherwise specified; *PTCL* peripheral T-cell lymphoma; *CTCL* cutaneous T-cell lymphoma; *HTCL* hepatosplenic T-cell lymphoma; *EATL* enteropathy-associated T-cell lymphoma; *MEITL* monomorphic epitheliotropic intestinal T-cell lymphoma; *BIA-ALCL* breast implant-associated ALCL; *ENKT* extranodal NK/T-cell lymphoma

Non-Hodgkin T-cell lymphomas (NHL-Ts) is a heterogeneous group of relatively rare malignancies with generally aggressive clinical behavior originating in T lymphocytes or natural killer (NK) cells. T-cell lymphomas (TCLs) account for 5–10% of all NHLs in Western countries, with an overall incidence of 0.5–2.0 per 100,000 inhabitants per year [115]. Depending on the histological subtype, NHL-T debuts with a nodal or extranodal presentation [116, 117]. The recent WHO and ICC classifications recognized the following subtypes as the primary nodal T and NK-derived neoplasias: follicular helper TCL (TFH); anaplastic large cell lymphoma (ALCL; ALK-positive and ALK-negative); peripheral TCL not otherwise specified (PTCL-NOS); and primary nodal EBV-positive T-/NK-cell lymphoma [1, 2, 118, 119]. Regarding extranodal subtypes, the most common extranodal entities TCL subtypes are the cutaneous TCL (CTCL), extranodal NK/T-cell lymphomas nasal type (ENKL), breast implant-associated anaplastic large cell lymphoma (BIA-ALCL), intestinal TCL (ITCL) and hepatosplenic TCL (HSTCL) [120] (Table 3 and Fig. 2).

**Table 3:** Summary of genetic alterations in T-cell lymphomas and their clinical utility

T-cell Lymphoma		Genetic alterations	Clinical significance	Refs.
Follicular helper T-cell lymphoma (TFH)	Angioimmunoblastic (AITL)	***IDH2***^***R172***^***, RHOA***^***G17V***^***, TET2, DNMT3A$,**** VAV1, CD28, ICOS, FYN* and *LCK* rearr	DIAGNOSTIC/PREDICTIVE^$^	[121, 122]
	Follicular type			
	Not otherwise specified (NOS)			
Anaplastic large cell lymphoma (ALCL)	ALK-positive	**ALK fusion,** NOTCH1*	DIAGNOSTIC/THERAPY*	[123, 125]
	ALK-negative	***DUSP22* rearr*#,**** JAK1, JAK3, STAT3*	DIAGNOSTIC/PROGNOSTIC^#^/PREDICTIVE	[127, 128]
		**Del(17p)/*TP53*, *TP63* rearr., *PRDM1* loss**	PROGNOSTIC	[128]
Peripheral T-cell lymphoma, not otherwise specified (PTCL-NOS)	PTCL-TBX21	***TET1, TET3, DNMT3A***	DIAGNOSTIC/PROGNOSTIC^#^	[126, 130]
	PTCL-GATA3	***TP53****#, PRDM1, CDKN2A/B, RB1* and *PTEN* loss*, STAT3 and MYC* gain		[120, 129, 130]
Primary nodal EBV-positive T-/NK-cell lymphoma		*TET2, PI3KCD, STAT3, TP53, CARD11*	DIAGNOSTIC	[131, 132]
Cutaneous T-cell lymphoma (CTCL)		***PLCG1,**** NFATC2, NFAT5, ZEB1, PRKCQ, RHOA, VAV1, PREX2, CTCF, ARID1A, TRRAP*	DIAGNOSTIC	[134, 136, 137, 139]
Hepatosplenic T-cell lymphoma (HTCL)		***STAT5B, STAT3, PIK3CD, SETD2, IN080, ARID1;*** Loss of *7p,* amplification of* 7q*	DIAGNOSTIC	[140, 141]
Breast implant-associated ALCL		***STAT3,**** JAK1, JAK3, DNMT3, TP53*	DIAGNOSTIC	[143–145]
Extranodal NK/T-cell lymphoma (ENKT), nasal type		***TP53#, DDX3X#,*** Del*(6q), STAT3, JAK3, STAT5B*	PROGNOSTIC^#^	[119]

The most relevant alterations are indicated in bold

*Del* deletion; *EBV* Epstein–Barr virus; *NK* natural killer; rear: rearrangement. *Refs*. references

*clinical significance

## Nodal T- and NK-cell lymphomas

### Follicular helper T‑cell lymphoma

Follicular helper T-cell (TFH) lymphoma comprises three entities with shared molecular features and a gene expression signature similar to TFH cells that define this subtype: the angioimmunoblastic (AITL), follicular, and NOS types. These lymphomas have similar mutational landscapes, including loss of function mutations in the methylation-associated genes *TET2* (in around 80% of cases) and *DNMT3A* (30–40%). Other recurrently mutated genes are *CD28*, *RHOA* (G17V), and *IDH2* (R172), primarily seen in a subset of AITLs, and genes in the TCR signaling pathway. *ICOS::CD28*, *ITK::SYK* and fusions involving *VAV1* are some of the recurrent fusions that have been described*.* In summary, TFH lymphomas share alterations in epigenetics and TCR signaling genes [118, 121, 122].

### Anaplastic large cell lymphoma

Anaplastic large cell lymphoma (ALCL) is characterized by pleomorphic tumor cells with uniform CD30 expression and includes four distinct subtypes [1, 2]: two nodal and two extranodal subtypes. This section will focus on systemic ALK-positive (with the *ALK* rearrangement, with several different fusion partners) and ALK-negative ALCL [118, 123, 124], which is more frequent in children and young adults. The most frequent *ALK* rearrangement is the t(2;5)(p23;q35), which leads to the fusion of nucleophosmin (NPM1) to ALK, resulting in a chimeric protein. These cases also have recurrent mutations in *NOTCH1*, which may represent a candidate therapy target [118, 124, 125]. Mutations in *TP53* and epigenetic regulators (*EP300, KMT2D/C*) have also been reported [126]. Immunophenotypic, histological, molecular, and clinical data are needed to diagnose ALK-positive ALCL correctly.

ALK-negative ALCL is quite a heterogeneous entity. The *DUSP22* rearrangement is present in around 20–30% of cases. Indeed, it has been defined as a distinct genetic ALK-negative subtype [127]. Around 60% of ALK-negative cases show activation of JAK-STAT3 through mutations mainly in *JAK1*, *JAK3*, and *STAT3*, or rearrangements involving *TYK2*, *ROS1*, and *FRK*. This activation is not present in ALK-negative ALCL with *DUSP22* rearrangements. A small percentage of ALK-negative ALCL cases feature the *TP63* rearrangement and losses of *TP53* and *PRDM1*, which are associated with an aggressive course [128].

### Peripheral T-cell lymphomas, PTCL-NOS

This group accounts for 34% of nodal PTCLs [117], including cases that do not meet the criteria for inclusion in the other defined entities. *FAT1* mutations have been reported to be frequent in PTCL-NOS and are associated with a worse prognosis [31, 129, 130]. This category is subdivided into two molecular subgroups, PTCL-TBX21 and PTCL-GATA3, which have different genetic landscapes [117]. It is characterized by high mortality and poor prognosis [31]. PTCL-GATA3 is more complex genetically and has a worse prognosis. It has frequent losses and mutations of *TP53* and *PRDM1*, losses of *CDKN2A/B*, *RB1*, and *PTEN* and gains of *STAT3* and *MYC*. PTCL-TBX21 shows frequent mutations in CMG, such as *TET1*, *TET3*, and *DNMT3A.*

### Primary nodal EBV-positive T-/NK-cell lymphoma

Primary nodal EBV-positive T-/NK-cell lymphoma is labeled a new provisional entity in ICC and WHO classifications [1, 2]. It is a rare disease of elderly or immunocompromised patients with poor prognosis. This entity shows low genomic instability, downregulation of EBV microRNAs, and frequent mutations in *STAT3, TET2*, *CARD11*, *BCOR*, *ARID1B*, *TP53*, and *PI3KCD* genes [118, 131, 132].

## Extranodal T- and NK-cell lymphomas

### Cutaneous T-cell lymphoma

Cutaneous T-cell lymphoma (CTCL) is a subtype of ectopic lymphoproliferative disease originating in the skin. The most common clinical manifestation is mycosis fungoides (MF), in which patients show cutaneous patches, plaques, or tumors [133]. Histologically, a CD4 + lymphocyte infiltration is observed. Sézary syndrome (SS) is a leukemic form of MF. Whole-genome, exome and targeted sequencing for SS and MF have revealed genomic alterations, including somatic mutations and CNAs), in genes that are well-known participants in key cellular activities like DNA damage (e.g., *TP53* (mut and del) and *ATM* (mainly del)), TCR signaling (*PLCG1, ZEB1* (del)), NF-κB signaling (*CARD11* and *TNFRSF1B*), CCR4/MAPK signaling (*CCR4*), JAK/STAT signaling (*JAK1/2/3, STAT3*, and *STAT5B*), cell migration (*RHOA*, the most common affecting p.N117, which is not found in other T-cell neoplasias, and *VAV1*), and chromatin remodeling (*ARID1A* and *CTCF*) [134–138]. Although most of them are not specific to CTCL diagnosis, some could be of theragnostic value, such as the *PLCG1* or JAK/STAT genes, and *RHOA* (p.N117I) seems to be CTCL-specific [139].

### T-cell lymphomas of the gastrointestinal tract

Intestinal T-cell lymphomas (ITCLs) comprise two main entities: enteropathy-associated TCL (EATL) and monomorphic epitheliotropic intestinal TCL (MEITL), as well as ITCL-NOS, diagnosed by exclusion and without specific clinicopathological characteristics (for a recent review see [120]).

EATL is more prevalent in Western populations and typically occurs in patients with celiac disease. It is characterized by recurring mutations in JAK/STAT pathway (*JAK1* and *STAT3*), NFkB (*TNFAIP3*), and epigenetic regulators (*KMT2D*, *BCOR* and *DDX3X*).

MEITL is a rare ITCL, and it is unrelated to celiac disease, affects older patients and is the main ITCL in Asia. The most prevalent mutations occur in *STAT5B* and *JAK3*, and mutually exclusive alterations in *BRAF*, *KRAS* and *NRAS* are detected more frequently than in EATL. Mutations in the epigenetic gene *SETD2* are also frequently detected in this entity.

### Hepatosplenic T-cell lymphoma

Hepatosplenic T-cell lymphoma (HSTCL) is a rare and aggressive TCL that affects adolescents and young adults. This group represents less than 1% of NHLs and is related to chronic immunosuppression [140]. Isochromosome (7q) can usually be detected, making it the most frequent chromosomal abnormality. Loss of 7p and amplification of 7q result in the altered expression of several oncogenes located on chromosome 7 (*CHN2*, *ABCB1*, *PPP1R9A*) [141]. Recent studies have detected genetic alterations that could be considered oncogenic drivers in the near future. *SETD2*, *IN080*, and *ARID1* mutations are involved in chromatin modification (occurring almost exclusively in HSTCL compared with other TCL subtypes). *STAT5B*, *STAT3*, and *PIK3CD* mutations have also been detected [140].

### Breast implant-associated ALCL

Breast implant-associated ALCL (BIA-ALCL) is a rare TCL that develops after a relatively long period following breast implant placement (8–11 years). It appears in fluids and capsules around the prosthesis. Its clinical, genomic, and molecular characteristics differ from other ALCLs [1]. They show clonal TCR rearrangements, and *STAT3* is recurrently mutated in up to 64% of BIA-ALCLs; other mutated genes include *JAK1*, *JAK3*, *DNMT3A*, and *TP53* [142–145]. *ALK*, *DUSP22,* and *TP63* rearrangements have not been found in BIA-ALCA cases [144, 146, 147].

### Extranodal NK/T‑cell lymphoma, nasal type

Extranodal NK/T‑cell lymphoma (ENKTL), nasal type comprises around 20–25% of mature T/NK-cell lymphomas in Asia and Central and South America, but only 5% in Europe and North America. It usually affects the upper aerodigestive tract. There is a strong association of ENKTL with EBV infection, although the mechanisms of its role are not fully understood. Deletion 6q21-25 is one of the most frequent genomic abnormalities, including *PRDM1*, *PTPRK,* and *FOXO3* genes. The most common mutations affect the JAK/STAT pathway (*STAT3*, *JAK3*, *STAT5B*), tumor suppressors genes (*TP53*, *DDX3X*), and epigenetic modifiers (*TET2* [~ 5–10%], *KMT2D*, *KMT2C*) [1, 2, 119, 120].

## Liquid biopsy in lymphomas

In recent years, high-throughput sequencing techniques have been successfully applied to the so-called “liquid biopsy” (LB), mainly to circulating tumor DNA (ctDNA). ctDNA represents the cell-free DNA released by the tumoral cell to body fluids, blood plasma being the most thoroughly analyzed form. The use of LBs, especially ctDNA, to support classical diagnostic tools in solid biopsy is increasing, mainly due to the advantages of this technique over the classic ones, as it is a minimally invasive approach that allows disease monitoring and detection of minimal residual disease (MRD) [148]. In fact, MRD detection in peripheral blood is a potent tool in various cancers, including hematological malignancies. Several studies have proved its clinical significance in DLBLC, cHL, MCL, PTCL [149–156], and, more recently, in indolent lymphomas, such as FL [157, 158].

Although the gold standard for mutational profiling is based on tissue biopsy, cfDNA genotyping would complement it. Moreover, it could be an excellent approach in certain situations, such as when dealing with inaccessible tumors or small tissue biopsies or when a re-biopsy is needed following relapse, transformation, or other clinical events. Concordance between ctDNA and FFPE in aggressive lymphomas is around 80% and somewhat lower in indolent lymphomas with low tumor burden [157]. However, even in these cases, ctDNA analysis has proved to be valid.

Basal or pre-treatment cfDNA analysis is valuable for tumor genotyping and as a surrogate for the tumor burden. The serial analysis allows the real-time follow-up of treatment response, clonal evolution monitoring, and MRD measurement [149–152, 157, 158]. For this reason, ctDNA genotyping may soon complement tissue analysis.

Additionally, the analysis of “in-phase” variants, done by two research groups who took slightly different approaches [159, 160], has improved the detection limit of ctDNA. This analysis tracks two or more somatic mutations in the same cfDNA fragment, lowering the background signal due to technical or biological errors. This approach is advantageous in lymphomas enriched in regions of aberrant SHM, leading to potentially more sensitive ctDNA detection and, thereby, greater MRD detection capacity [157, 159, 160].

However, efforts must still be made to standardize every step to successfully apply LB in the clinical milieu, from sample manipulation to bioinformatic analyses [148, 161, 162]. These technical and analytical considerations are serious challenges and are the focus of multiple cooperative efforts to allow its eventual application in routine clinical practice.

## Conclusions

Thanks to the development of genomics, molecular data have enabled the better diagnosis of lymphomas, and these alterations are now part of the diagnostic criteria. NGS allows the simultaneous detection of multiple alterations, including mutations, copy number alterations and structural aberrations, and this genomic information may be combined with morphology and immunophenotyping for the purposes of diagnosis and prognosis. However, standardization throughout the entire process and quality controls are prerequisites for NGS implementation in lymphoma diagnosis. The panels proposed in this review (Table 4) for SNVs and CNAs detection are intended to be a helpful tool to encourage the implementation of NGS-based lymphoma diagnosis.

**Table 4:** Proposed next-generation sequencing panels for B- and T-cell lymphomas

B-cell lymphomas							T-cell lymphomas						
Mandatory		Recommended		CNAs			Mandatory		Recommended		CNAs		
*B2M*	*KTM2D*	*ARID1A*	*MEF2B*	*ATM*	CN gain	11p	*CD28*	*RHOA*	*ARID1A*	*NOTCH1*	*TP53*	CN loss	17p13.1
*BCL10*	*MYC*	*ARID1B*	*MKLN1*	*BCL2*	CN gain	18q21.33	*DNMT3A*	*SETD2*	*CTCF*	*PREX2*	*MYC*	CN gain	8q24.21
*BCL2*	*MYD88*	*ATM*	*NFKBIE*	*BCL6*	CN gain	3q27.3	*IDH2*	*STAT3*	*FYN*	*PRKCQ*	*CDKN2A*	CN loss	9p21.3
*BCL6*	*NOTCH1*	*CD79A*	*NSD2*	*CDKN2A*	CN loss	9p21.3	*JAK1*	*STAT5B*	*IN080*	*RB1*			
*BIRC3*	*NOTCH2*	*CSFR2B*	*P2RY8*	*FOXO1*	CN gain	13q	*JAK3*	*TET1*	*LCK*	*TET3*			
*BTG1*	*PIM1*	*DDX3X*	*PLEKHG1*	*MYC*	CN gain	8q24.21	*PI3KCD*	*TET2*	*NFAT5*	*TRRAP*			
*BTK*	*PIM2*	*FBXW7*	*POT1*	*TP53*	CN loss	17p13.1	*PLCG1*	*TP53*	*NFATC2*				
*CARD11*	*PLCG2*	*FOXO1*	*PTPN11*	*CCND1*	CN gain	11q13	*PRDM1*	*VAV1*					
*CCND3*	*POU2AF1*	*HIVEP2*	*S1PR2*	*Del (7q)*	CN loss	7q31–32							
*CD58*	*PRDM1*	*IGLL5*	*SIN3A*	*TRISOMY 3*	CN gain								
*CD70*	*SF3B1*	*IKBK*	*RPS15*	*TRISOMY 12*	CN gain								
*CD79B*	*SGK1*	*IKZF3*	*TRAF2*	*TRISOMY 18*	CN gain								
*CHD2*	*SOCS1*	*ITPKB*	*TRAF3*										
*CREBBP*	*STAT3*	*LYN*	*XPO1*										
*DTX1*	*STAT6*	*MAP2K1*	*ZMYM3*										
*EP300*	*TCF3*	*MAP3K14*	*ZNF292*										
*EZH2*	*TET2*												
*GNA13*	*TNFAIP3*												
*HIST1H1E*	*TNFRSF14*												
*IRF8*	*TP53*												
*ID3*	*UBE2A*												
*KLF2*	IGHV												

*CNAs* Copy number alterations; *CN* Copy number

## Data Availability

Not applicable.

## References

[1] Campo E, Jaffe ES, Cook JR, Quintanilla-Martinez L, Swerdlow SH, Anderson KC (2022). The International Consensus Classification of Mature Lymphoid Neoplasms: a report from the clinical advisory committee. Blood.

[2] Alaggio R, Amador C, Anagnostopoulos I, Attygalle AD, de Araujo IBO, Berti E (2022). The 5th of the World Health Organization Classification of Haematolymphoid Tumours: lymphoid neoplasms. Leukemia.

[3] Rozman C, Montserrat E (1995). Chronic lymphocytic leukemia. N Engl J Med.

[4] Non-Hodgkin Lymphoma—Cancer Stat Facts. SEER. [cited 2021 Jun 9].

[5] Hallek M, Al-Sawaf O (2021). Chronic lymphocytic leukemia: 2022 update on diagnostic and therapeutic procedures. Am J Hematol.

[6] Döhner H, Stilgenbauer S, Benner A, Leupolt E, Kröber A, Bullinger L (2000). Genomic aberrations and survival in chronic lymphocytic leukemia. N Engl J Med.

[7] Tari K, Shamsi Z, Reza Ghafari H, Atashi A, Shahjahani M, Abroun S (2018). The role of the genetic abnormalities, epigenetic and microRNA in the prognosis of chronic lymphocytic leukemia. Exp Oncol.

[8] González-Gascón Y, Marín I, Hernández-Sánchez M, Rodríguez-Vicente A-E, Sanzo C, Aventín A, Puiggros A (2016). A high proportion of cells carrying trisomy 12 is associated with a worse outcome in patients with chronic lymphocytic leukemia. Hematol Oncol.

[9] Döhner H, Stilgenbauer S, James MR, Benner A, Weilguni T, Bentz M (1997). 11q deletions identify a new subset of B-cell chronic lymphocytic leukemia characterized by extensive nodal involvement and inferior prognosis. Blood.

[10] Bagacean C, Tempescul A, Ternant D, Banet A, Douet-Guilbert N, Bordron A (2019). 17p deletion strongly influences rituximab elimination in chronic lymphocytic leukemia. J Immunother Cancer.

[11] O’Brien S, Jones JA, Coutre SE, Mato AR, Hillmen P, Tam C (2016). Ibrutinib for patients with relapsed or refractory chronic lymphocytic leukaemia with 17p deletion (RESONATE-17): a phase 2, open-label, multicentre study. Lancet Oncol.

[12] Stilgenbauer S, Eichhorst B, Schetelig J, Hillmen P, Seymour JF, Coutre S (2018). Venetoclax for patients with chronic lymphocytic leukemia with 17p deletion: results from the full population of a phase II pivotal trial. J Clin Oncol.

[13] Jondreville L, Krzisch D, Chapiro E, Nguyen-Khac F (2020). The complex karyotype and chronic lymphocytic leukemia: prognostic value and diagnostic recommendations. Am J Hematol.

[14] Damle RN, Wasil T, Fais F, Ghiotto F, Valetto A, Allen SL (1999). Ig V gene mutation status and CD38 expression as novel prognostic indicators in chronic lymphocytic leukemia. Blood.

[15] Crombie J, Davids MS (2017). IGHV mutational status testing in chronic lymphocytic leukemia. Am J Hematol.

[16] Nadeu F, Royo R, Clot G, Duran-Ferrer M, Navarro A, Martín S (2021). IGLV3-21R110 identifies an aggressive biological subtype of chronic lymphocytic leukemia with intermediate epigenetics. Blood.

[17] Rossi D, Cerri M, Deambrogi C, Sozzi E, Cresta S, Rasi S (2009). The prognostic value of TP53 mutations in chronic lymphocytic leukemia is independent of Del17p13: implications for overall survival and chemorefractoriness. Clin Cancer Res.

[18] Zenz T, Vollmer D, Trbusek M, Smardova J, Benner A, Soussi T (2010). TP53 mutation profile in chronic lymphocytic leukemia: evidence for a disease specific profile from a comprehensive analysis of 268 mutations. Leukemia.

[19] Landau DA, Carter SL, Stojanov P, McKenna A, Stevenson K, Lawrence MS (2013). Evolution and impact of subclonal mutations in chronic lymphocytic leukemia. Cell.

[20] González-Rincón J, Gómez S, Martinez N, Troulé K, Perales-Patón J, Derdak S (2019). Clonal dynamics monitoring during clinical evolution in chronic lymphocytic leukaemia. Sci Rep.

[21] González-Rincón J, Garcia-Vela JA, Gómez S, Fernández-Cuevas B, Nova-Gurumeta S, Pérez-Sanz N (2021). Genomic mutation profile in progressive chronic lymphocytic leukemia patients prior to first-line chemoimmunotherapy with FCR and rituximab maintenance (REM). PLoS ONE.

[22] Doménech E, Gómez-López G, Gzlez-Peña D, López M, Herreros B, Menezes J (2012). New mutations in chronic lymphocytic leukemia identified by target enrichment and deep sequencing. PLoS ONE.

[23] Fabbri G, Rasi S, Rossi D, Trifonov V, Khiabanian H, Ma J (2011). Analysis of the chronic lymphocytic leukemia coding genome: role of NOTCH1 mutational activation. J Exp Med.

[24] Fabbri G, Khiabanian H, Holmes AB, Wang J, Messina M, Mullighan CG (2013). Genetic lesions associated with chronic lymphocytic leukemia transformation to Richter syndrome. J Exp Med.

[25] Chigrinova E, Rinaldi A, Kwee I, Rossi D, Rancoita PMV, Strefford JC (2013). Two main genetic pathways lead to the transformation of chronic lymphocytic leukemia to Richter syndrome. Blood.

[26] Xu L, Tsakmaklis N, Yang G, Chen JG, Liu X, Demos M (2017). Acquired mutations associated with ibrutinib resistance in Waldenström macroglobulinemia. Blood.

[27] Quinquenel A, Fornecker L-M, Letestu R, Ysebaert L, Fleury C, Lazarian G (2019). Prevalence of BTK and PLCG2 mutations in a real-life CLL cohort still on ibrutinib after 3 years: a FILO group study. Blood.

[28] Tausch E, Close W, Dolnik A, Bloehdorn J, Chyla B, Bullinger L (2019). Venetoclax resistance and acquired BCL2 mutations in chronic lymphocytic leukemia. Haematologica.

[29] Castillo JJ, Ghobrial IM, Treon SP (2015). Biology, prognosis, and therapy of Waldenström macroglobulinemia. Cancer Treat Res.

[30] Ganapathi KA, Brown LE, Prakash S, Bhargava P (2021). New developments in non-Hodgkin lymphoid malignancies. Pathology.

[31] de Leval L, Alizadeh AA, Bergsagel PL, Campo E, Davies A, Dogan A (2022). Genomic profiling for clinical decision making in lymphoid neoplasms. Blood.

[32] Treon SP, Tripsas CK, Meid K, Warren D, Varma G, Green R (2015). Ibrutinib in previously treated Waldenström’s macroglobulinemia. N Engl J Med.

[33] Kaiser LM, Hunter ZR, Treon SP, Buske C (2021). CXCR4 in Waldenström’s macroglobulinema: chances and challenges. Leukemia.

[34] Wang Y, Gali VL, Xu-Monette ZY, Sano D, Thomas SK, Weber DM (2021). Molecular and genetic biomarkers implemented from next-generation sequencing provide treatment insights in clinical practice for Waldenström macroglobulinemia. Neoplasia.

[35] Zucca E, Arcaini L, Buske C, Johnson PW, Ponzoni M, Raderer M (2020). Marginal zone lymphomas: ESMO clinical practice guidelines for diagnosis, treatment and follow-up. Ann Oncol.

[36] Rossi D, Bertoni F, Zucca E (2022). Marginal-zone lymphomas. N Engl J Med.

[37] Bonfiglio F, Bruscaggin A, Guidetti F, Di Terzi Bergamo L, Faderl M, Spina V (2022). Genetic and phenotypic attributes of splenic marginal zone lymphoma. Blood.

[38] Thieblemont C (2017). Improved biological insight and influence on management in indolent lymphoma. Talk 3: update on nodal and splenic marginal zone lymphoma. Hematol Am Soc Hematol Educ Program.

[39] Mateo M, Mollejo M, Villuendas R, Algara P, Sanchez-Beato M, Martínez P (1999). 7q31-32 allelic loss is a frequent finding in splenic marginal zone lymphoma. Am J Pathol.

[40] Mateo M-S, Mollejo M, Villuendas R, Algara P, Sanchez-Beato M, Martínez P (2001). Molecular heterogeneity of splenic marginal zone lymphomas: analysis of mutations in the 5? Non-coding region of the bcl-6 gene. Leukemia.

[41] Vela V, Juskevicius D, Dirnhofer S, Menter T, Tzankov A (2022). Mutational landscape of marginal zone B-cell lymphomas of various origin: organotypic alterations and diagnostic potential for assignment of organ origin. Virchows Arch.

[42] Zucca E, Bertoni F (2016). The spectrum of MALT lymphoma at different sites: biological and therapeutic relevance. Blood.

[43] Du M-Q (2017). MALT lymphoma: genetic abnormalities, immunological stimulation and molecular mechanism. Best Pract Res Clin Haematol.

[44] Novak U, Rinaldi A, Kwee I, Nandula SV, Rancoita PMV, Compagno M (2009). The NF-{kappa}B negative regulator TNFAIP3 (A20) is inactivated by somatic mutations and genomic deletions in marginal zone lymphomas. Blood.

[45] Conconi A, Martinelli G, Lopez-Guillermo A, Zinzani PL, Ferreri AJM, Rigacci L (2011). Clinical activity of bortezomib in relapsed/refractory MALT lymphomas: results of a phase II study of the international extranodal lymphoma study group (IELSG). Ann Oncol.

[46] Bachy E, Seymour JF, Feugier P, Offner F, López-Guillermo A, Belada D (2019). Sustained progression-free survival benefit of rituximab maintenance in patients with follicular lymphoma: long-term results of the PRIMA study. J Clin Oncol.

[47] Leich E, Salaverria I, Bea S, Zettl A, Wright G, Moreno V (2009). Follicular lymphomas with and without translocation t(14;18) differ in gene expression profiles and genetic alterations. Blood.

[48] Nann D, Ramis-Zaldivar JE, Müller I, Gonzalez-Farre B, Schmidt J, Egan C (2020). Follicular lymphoma t(14;18)-negative is genetically a heterogeneous disease. Blood Adv.

[49] Hellmuth JC, Louissaint A, Szczepanowski M, Haebe S, Pastore A, Alig S (2018). Duodenal-type and nodal follicular lymphomas differ by their immune microenvironment rather than their mutation profiles. Blood.

[50] Schmidt J, Ramis-Zaldivar JE, Nadeu F, Gonzalez-Farre B, Navarro A, Egan C (2017). Mutations of MAP2K1 are frequent in pediatric-type follicular lymphoma and result in ERK pathway activation. Blood.

[51] Morin RD, Mendez-Lago M, Mungall AJ, Goya R, Mungall KL, Corbett RD (2011). Frequent mutation of histone-modifying genes in non-Hodgkin lymphoma. Nature.

[52] Green MR, Gentles AJ, Nair RV, Irish JM, Kihira S, Liu CL (2013). Hierarchy in somatic mutations arising during genomic evolution and progression of follicular lymphoma. Blood.

[53] Green MR, Kihira S, Liu CL, Nair RV, Salari R, Gentles AJ (2015). Mutations in early follicular lymphoma progenitors are associated with suppressed antigen presentation. Proc Natl Acad Sci USA.

[54] Apostolidis J, Mokhtar N, Al Omari R, Darweesh M, Al HH (2020). Follicular lymphoma: update on management and emerging therapies at the dawn of the new decade. Hematol Oncol.

[55] Morschhauser F, Tilly H, Chaidos A, McKay P, Phillips T, Assouline S (2020). Tazemetostat for patients with relapsed or refractory follicular lymphoma: an open-label, single-arm, multicentre, phase 2 trial. Lancet Oncol.

[56] Pastore A, Jurinovic V, Kridel R, Hoster E, Staiger AM, Szczepanowski M (2015). Integration of gene mutations in risk prognostication for patients receiving first-line immunochemotherapy for follicular lymphoma: a retrospective analysis of a prospective clinical trial and validation in a population-based registry. Lancet Oncol.

[57] Lockmer S, Ren W, Brodtkorb M, Østenstad B, Wahlin BE, Pan-Hammarström Q (2020). M7-FLIPI is not prognostic in follicular lymphoma patients with first-line rituximab chemo-free therapy. Br J Haematol.

[58] Pasqualucci L, Khiabanian H, Fangazio M, Vasishtha M, Messina M, Holmes AB (2014). Genetics of follicular lymphoma transformation. Cell Rep.

[59] Okosun J, Bödör C, Wang J, Araf S, Yang C-Y, Pan C (2014). Integrated genomic analysis identifies recurrent mutations and evolution patterns driving the initiation and progression of follicular lymphoma. Nat Genet.

[60] Kridel R, Chan FC, Mottok A, Boyle M, Farinha P, Tan K (2016). Histological transformation and progression in follicular lymphoma: a clonal evolution study. PLoS Med.

[61] González-Rincón J, Méndez M, Gómez S, García JF, Martín P, Bellas C (2019). Unraveling transformation of follicular lymphoma to diffuse large B-cell lymphoma. PLoS ONE.

[62] Puente XS, Jares P, Campo E (2018). Chronic lymphocytic leukemia and mantle cell lymphoma: crossroads of genetic and microenvironment interactions. Blood.

[63] Navarro A, Beà S, Jares P, Campo E (2020). Molecular pathogenesis of mantle cell lymphoma. Hematol Oncol Clin North Am.

[64] Beà S, Valdés-Mas R, Navarro A, Salaverria I, Martín-Garcia D, Jares P (2013). Landscape of somatic mutations and clonal evolution in mantle cell lymphoma. Proc Natl Acad Sci USA.

[65] Ferrero S, Rossi D, Rinaldi A, Bruscaggin A, Spina V, Eskelund CW (2020). KMT2D mutations and TP53 disruptions are poor prognostic biomarkers in mantle cell lymphoma receiving high-dose therapy: a FIL study. Haematologica.

[66] Jain P, Wang ML (2022). Mantle cell lymphoma in 2022-A comprehensive update on molecular pathogenesis, risk stratification, clinical approach, and current and novel treatments. Am J Hematol.

[67] Wu C, de Miranda NF, Chen L, Wasik AM, Mansouri L, Jurczak W (2016). Genetic heterogeneity in primary and relapsed mantle cell lymphomas: impact of recurrent CARD11 mutations. Oncotarget.

[68] Jain P, Kanagal-Shamanna R, Zhang S, Ahmed M, Ghorab A, Zhang L (2018). Long-term outcomes and mutation profiling of patients with mantle cell lymphoma (MCL) who discontinued ibrutinib. Br J Haematol.

[69] Ramis-Zaldivar JE, Gonzalez-Farré B, Balagué O, Celis V, Nadeu F, Salmerón-Villalobos J (2020). Distinct molecular profile of IRF4-rearranged large B-cell lymphoma. Blood.

[70] Alizadeh AA, Eisen MB, Davis RE, Ma C, Lossos IS, Rosenwald A (2000). Distinct types of diffuse large B-cell lymphoma identified by gene expression profiling. Nature.

[71] Coiffier B, Thieblemont C, Neste EVD, Lepeu G, Plantier I, Castaigne S (2010). Long-term outcome of patients in the LNH-98.5 trial, the first randomized study comparing rituximab-CHOP to standard CHOP chemotherapy in DLBCL patients: a study by the Groupe d’Etudes des lymphomes de l’Adulte. Blood.

[72] Pérez-Callejo D, González-Rincón J, Sánchez A, Provencio M, Sánchez-Beato M (2015). Action and resistance of monoclonal CD20 antibodies therapy in B-cell non-Hodgkin lymphomas. Cancer Treat Rev.

[73] Schmitz R, Wright GW, Huang DW, Johnson CA, Phelan JD, Wang JQ (2018). Genetics and pathogenesis of diffuse large B-cell lymphoma. N Engl J Med.

[74] Wright GW, Huang DW, Phelan JD, Coulibaly ZA, Roulland S, Young RM (2020). A probabilistic classification tool for genetic subtypes of diffuse large B cell lymphoma with therapeutic implications. Cancer Cell.

[75] Chapuy B, Stewart C, Dunford AJ, Kim J, Kamburov A, Redd RA (2018). Molecular subtypes of diffuse large B cell lymphoma are associated with distinct pathogenic mechanisms and outcomes. Nat Med.

[76] Lacy SE, Barrans SL, Beer PA, Painter D, Smith AG, Roman E (2020). Targeted sequencing in DLBCL, molecular subtypes, and outcomes: a haematological malignancy research network report. Blood.

[77] Pedrosa L, Fernández-Miranda I, Pérez-Callejo D, Quero C, Rodríguez M, Martín-Acosta P (2021). Proposal and validation of a method to classify genetic subtypes of diffuse large B cell lymphoma. Sci Rep.

[78] Mishina T, Oshima-Hasegawa N, Tsukamoto S, Fukuyo M, Kageyama H, Muto T (2021). Genetic subtype classification using a simplified algorithm and mutational characteristics of diffuse large B-cell lymphoma in a Japanese cohort. Br J Haematol.

[79] Franco F, González-Rincón J, Lavernia J, García JF, Martín P, Bellas C (2017). Mutational profile of primary breast diffuse large B-cell lymphoma. Oncotarget.

[80] Gebauer N, Künstner A, Ketzer J, Witte HM, Rausch T, Benes V (2021). Genomic insights into the pathogenesis of Epstein-Barr virus-associated diffuse large B-cell lymphoma by whole-genome and targeted amplicon sequencing. Blood Cancer J.

[81] Kataoka K, Miyoshi H, Sakata S, Dobashi A, Couronné L, Kogure Y (2019). Frequent structural variations involving programmed death ligands in Epstein-Barr virus-associated lymphomas. Leukemia.

[82] Randall C, Fedoriw Y (2020). Pathology and diagnosis of follicular lymphoma and related entities. Pathology.

[83] Wagener R, Seufert J, Raimondi F, Bens S, Kleinheinz K, Nagel I (2019). The mutational landscape of Burkitt-like lymphoma with 11q aberration is distinct from that of Burkitt lymphoma. Blood.

[84] Olszewski AJ, Kurt H, Evens AM (2022). Defining and treating high-grade B-cell lymphoma. NOS Blood.

[85] Ennishi D, Jiang A, Boyle M, Collinge B, Grande BM, Ben-Neriah S (2019). Double-hit gene expression signature defines a distinct subgroup of germinal center B-cell-like diffuse large B-cell lymphoma. J Clin Oncol.

[86] Sha C, Barrans S, Cucco F, Bentley MA, Care MA, Cummin T (2019). Molecular high-grade B-cell lymphoma: defining a poor-risk group that requires different approaches to therapy. J Clin Oncol.

[87] Cucco F, Barrans S, Sha C, Clipson A, Crouch S, Dobson R (2020). Distinct genetic changes reveal evolutionary history and heterogeneous molecular grade of DLBCL with MYC/BCL2 double-hit. Leukemia.

[88] Roschewski M, Staudt LM, Wilson WH (2022). Burkitt’s lymphoma. New Engl J Med.

[89] Blum KA, Lozanski G, Byrd JC (2004). Adult Burkitt leukemia and lymphoma. Blood.

[90] Ma MCJ, Tadros S, Bouska A, Heavican T, Yang H, Deng Q (2022). Subtype-specific and co-occurring genetic alterations in B-cell non-Hodgkin lymphoma. Haematologica.

[91] Panea RI, Love CL, Shingleton JR, Reddy A, Bailey JA, Moormann AM (2019). The whole-genome landscape of Burkitt lymphoma subtypes. Blood.

[92] Schmitz R, Young RM, Ceribelli M, Jhavar S, Xiao W, Zhang M (2012). Burkitt lymphoma pathogenesis and therapeutic targets from structural and functional genomics. Nature.

[93] Dave SS, Fu K, Wright GW, Lam LT, Kluin P, Boerma E-J (2006). Molecular diagnosis of Burkitt’s lymphoma. New Engl J Med.

[94] Hummel M, Bentink S, Berger H, Klapper W, Wessendorf S, Barth TFE (2006). A biologic definition of Burkitt’s lymphoma from transcriptional and genomic profiling. New Engl J Med.

[95] Piccaluga PP, Falco GD, Kustagi M, Gazzola A, Agostinelli C, Tripodo C (2011). Gene expression analysis uncovers similarity and differences among Burkitt lymphoma subtypes. Blood.

[96] Newman AM, Zaka M, Zhou P, Blain AE, Erhorn A, Barnard A (2022). Genomic abnormalities of TP53 define distinct risk groups of paediatric B-cell non-Hodgkin lymphoma. Leukemia.

[97] Gong C, Krupka JA, Gao J, Grigoropoulos NF, Giotopoulos G, Asby R (2021). Sequential inverse dysregulation of the RNA helicases DDX3X and DDX3Y facilitates MYC-driven lymphomagenesis. Mol Cell.

[98] Muppidi JR, Schmitz R, Green JA, Xiao W, Larsen AB, Braun SE (2014). Loss of signalling via G α 13 in germinal centre B-cell-derived lymphoma. Nature.

[99] Kabrani E, Chu VT, Tasouri E, Sommermann T, Baßler K, Ulas T (2018). Nuclear FOXO1 promotes lymphomagenesis in germinal center B cells. Blood.

[100] Grande BM, Gerhard DS, Jiang A, Griner NB, Abramson JS, Alexander TB (2019). Genome-wide discovery of somatic coding and noncoding mutations in pediatric endemic and sporadic Burkitt lymphoma. Blood.

[101] Leoncini L (2022). Epstein-Barr virus positivity as a defining pathogenetic feature of Burkitt lymphoma subtypes. Br J Haematol.

[102] López C, Kleinheinz K, Aukema SM, Rohde M, Bernhart SH, Hübschmann D (2019). Genomic and transcriptomic changes complement each other in the pathogenesis of sporadic Burkitt lymphoma. Nat Commun.

[103] Hartmann S, Eichenauer DA (2020). Nodular lymphocyte predominant Hodgkin lymphoma: pathology, clinical course and relation to T-cell/histiocyte rich large B-cell lymphoma. Pathology.

[104] Joos S, Menz CK, Wrobel G, Siebert R, Gesk S, Ohl S (2002). Classical Hodgkin lymphoma is characterized by recurrent copy number gains of the short arm of chromosome 2. Blood.

[105] Martín-Subero JI, Gesk S, Harder L, Sonoki T, Tucker PW, Schlegelberger B (2002). Recurrent involvement of the REL and BCL11A loci in classical Hodgkin lymphoma. Blood.

[106] Steidl C, Telenius A, Shah SP, Farinha P, Barclay L, Boyle M (2010). Genome-wide copy number analysis of Hodgkin Reed-Sternberg cells identifies recurrent imbalances with correlations to treatment outcome. Blood.

[107] Green MR, Monti S, Rodig SJ, Juszczynski P, Currie T, O’Donnell E (2010). Integrative analysis reveals selective 9p24.1 amplification, increased PD-1 ligand expression, and further induction via JAK2 in nodular sclerosing Hodgkin lymphoma and primary mediastinal large B-cell lymphoma. Blood.

[108] Mata E, Fernández S, Astudillo A, Fernández R, García-Cosío M, Sánchez-Beato M (2019). Genomic analyses of microdissected Hodgkin and Reed-Sternberg cells: mutations in epigenetic regulators and p53 are frequent in refractory classic Hodgkin lymphoma. Blood Cancer J.

[109] Mata E, Díaz-López A, Martín-Moreno AM, Sánchez-Beato M, Varela I, Mestre MJ (2017). Analysis of the mutational landscape of classic Hodgkin lymphoma identifies disease heterogeneity and potential therapeutic targets. Oncotarget.

[110] Reichel J, Chadburn A, Rubinstein PG, Giulino-Roth L, Tam W, Liu Y (2015). Flow sorting and exome sequencing reveal the oncogenome of primary Hodgkin and Reed-Sternberg cells. Blood.

[111] Tiacci E, Ladewig E, Schiavoni G, Penson A, Fortini E, Pettirossi V (2018). Pervasive mutations of JAK-STAT pathway genes in classical Hodgkin lymphoma. Blood.

[112] Steidl C, Shah SP, Woolcock BW, Rui L, Kawahara M, Farinha P (2011). MHC class II transactivator CIITA is a recurrent gene fusion partner in lymphoid cancers. Nature.

[113] Montalban C, Abraira V, Morente M, Acevedo A, Aguilera B, Bellas C (2000). Epstein-Barr virus-latent membrane protein 1 expression has a favorable influence in the outcome of patients with Hodgkin’s disease treated with chemotherapy. Leuk Lymphoma.

[114] Weniger MA, Küppers R (2021). Molecular biology of Hodgkin lymphoma. Leukemia.

[115] Bellei M, Chiattone CS, Luminari S, Pesce EA, Cabrera ME, de Souza CA (2012). T-cell lymphomas in South America and europe. Rev Bras Hematol Hemoter.

[116] Horwitz SM, Ansell S, Ai WZ, Barnes J, Barta SK, Brammer J (2022). T-cell lymphomas, version 2.2022, NCCN clinical practice guidelines in oncology. J Natl Compr Canc Netw.

[117] d’Amore F, Gaulard P, Trümper L, Corradini P, Kim W-S, Specht L (2015). Peripheral T-cell lymphomas: ESMO clinical practice guidelines for diagnosis, treatment and follow-up. Ann Oncol.

[118] Feldman AL, Laurent C, Narbaitz M, Nakamura S, Chan WC, de Leval L (2023). Classification and diagnostic evaluation of nodal T- and NK-cell lymphomas. Virchows Arch.

[119] Lewis NE, Sardana R, Dogan A (2023). Mature T-cell and NK-cell lymphomas: updates on molecular genetic features. Int J Hematol.

[120] de Leval L, Feldman AL, Pileri S, Nakamura S, Gaulard P (2023). Extranodal T- and NK-cell lymphomas. Virchows Arch.

[121] Dobay MP, Lemonnier F, Missiaglia E, Bastard C, Vallois D, Jais J-P (2017). Integrative clinicopathological and molecular analyses of angioimmunoblastic T-cell lymphoma and other nodal lymphomas of follicular helper T-cell origin. Haematologica.

[122] Streubel B, Vinatzer U, Willheim M, Raderer M, Chott A (2006). Novel t(5;9)(q33;q22) fuses ITK to SYK in unspecified peripheral T-cell lymphoma. Leukemia.

[123] Benharroch D, Meguerian-Bedoyan Z, Lamant L, Amin C, Brugières L, Terrier-Lacombe MJ (1998). ALK-positive lymphoma: a single disease with a broad spectrum of morphology. Blood.

[124] Parrilla Castellar ER, Jaffe ES, Said JW, Swerdlow SH, Ketterling RP, Knudson RA (2014). ALK-negative anaplastic large cell lymphoma is a genetically heterogeneous disease with widely disparate clinical outcomes. Blood.

[125] Larose H, Prokoph N, Matthews JD, Schlederer M, Högler S, Alsulami AF (2021). Whole exome sequencing reveals NOTCH1 mutations in anaplastic large cell lymphoma and points to Notch both as a key pathway and a potential therapeutic target. Haematologica.

[126] Lobello C, Tichy B, Bystry V, Radova L, Filip D, Mraz M (2021). STAT3 and TP53 mutations associate with poor prognosis in anaplastic large cell lymphoma. Leukemia.

[127] King RL, Dao LN, McPhail ED, Jaffe ES, Said J, Swerdlow SH (2016). Morphologic features of ALK-negative anaplastic large cell lymphomas with DUSP22 rearrangements. Am J Surg Pathol.

[128] Karube K, Feldman AL (2020). “Double-hit” of DUSP22 and TP63 rearrangements in anaplastic large cell lymphoma. ALK-negative Blood.

[129] Heavican TB, Bouska A, Yu J, Lone W, Amador C, Gong Q (2019). Genetic drivers of oncogenic pathways in molecular subgroups of peripheral T-cell lymphoma. Blood.

[130] Laginestra MA, Cascione L, Motta G, Fuligni F, Agostinelli C, Rossi M (2020). Whole exome sequencing reveals mutations in FAT1 tumor suppressor gene clinically impacting on peripheral T-cell lymphoma not otherwise specified. Mod Pathol.

[131] Wai CMM, Chen S, Phyu T, Fan S, Leong SM, Zheng W (2022). Immune pathway upregulation and lower genomic instability distinguish EBV-positive nodal T/NK-cell lymphoma from ENKTL and PTCL-NOS. Haematologica.

[132] Gonzalez Barca E, Tomás-Roca L, Esteve A, Rodriguez M, Gato L, Alonso-Alonso R (2023). Extranodal natural killer/T-cell lymphoma nasal type in a western population: molecular profiling identifies new therapeutic targets. Am J Hematol.

[133] García-Díaz N, Piris MÁ, Ortiz-Romero PL, Vaqué JP (2021). Mycosis fungoides and Sézary syndrome: an integrative review of the pathophysiology, molecular drivers, and targeted therapy. Cancers.

[134] Choi J, Goh G, Walradt T, Hong BS, Bunick CG, Chen K (2015). Genomic landscape of cutaneous T cell lymphoma. Nat Genet.

[135] Park J, Daniels J, Wartewig T, Ringbloom KG, Martinez-Escala ME, Choi S (2021). Integrated genomic analyses of cutaneous T cell lymphomas reveal the molecular bases for disease heterogeneity. Blood.

[136] Vaqué JP, Gómez-López G, Monsálvez V, Varela I, Martínez N, Pérez C (2014). PLCG1 mutations in cutaneous T-cell lymphomas. Blood.

[137] da Silva Almeida AC, Abate F, Khiabanian H, Martinez-Escala E, Guitart J, Tensen CP (2015). The mutational landscape of cutaneous T cell lymphoma and Sézary syndrome. Nat Genet.

[138] Prasad A, Rabionet R, Espinet B, Zapata L, Puiggros A, Melero C (2016). Identification of gene mutations and fusion genes in patients with Sézary syndrome. J Invest Dermatol.

[139] Park J, Yang J, Wenzel AT, Ramachandran A, Lee WJ, Daniels JC (2017). Genomic analysis of 220 CTCLs identifies a novel recurrent gain-of-function alteration in RLTPR (p.Q575E). Blood.

[140] Yabe M, Miranda RN, Medeiros LJ (2018). Hepatosplenic T-cell lymphoma: a review of clinicopathologic features, pathogenesis, and prognostic factors. Hum Pathol.

[141] McKinney M, Moffitt AB, Gaulard P, Travert M, De Leval L, Nicolae A (2017). The genetic basis of hepatosplenic T-cell lymphoma. Cancer Discov.

[142] McCarthy CM, Loyo-Berríos N, Qureshi AA, Mullen E, Gordillo G, Pusic AL (2019). Patient registry and outcomes for breast implants and anaplastic large cell lymphoma etiology and epidemiology (PROFILE): initial report of findings, 2012–2018. Plast Reconstr Surg.

[143] Blombery P, Thompson ER, Jones K, Arnau GM, Lade S, Markham JF (2016). Whole exome sequencing reveals activating JAK1 and STAT3 mutations in breast implant-associated anaplastic large cell lymphoma anaplastic large cell lymphoma. Haematologica.

[144] Oishi N, Brody GS, Ketterling RP, Viswanatha DS, He R, Dasari S (2018). Genetic subtyping of breast implant-associated anaplastic large cell lymphoma. Blood.

[145] Letourneau A, Maerevoet M, Milowich D, Dewind R, Bisig B, Missiaglia E (2018). Dual JAK1 and STAT3 mutations in a breast implant-associated anaplastic large cell lymphoma. Virchows Arch.

[146] Gerbe A, Alame M, Dereure O, Gonzalez S, Durand L, Tempier A (2019). Systemic, primary cutaneous, and breast implant-associated ALK-negative anaplastic large-cell lymphomas present similar biologic features despite distinct clinical behavior. Virchows Arch.

[147] Laurent C, Delas A, Gaulard P, Haioun C, Moreau A, Xerri L (2016). Breast implant-associated anaplastic large cell lymphoma: two distinct clinicopathological variants with different outcomes. Ann Oncol.

[148] Huet S, Salles G (2020). Potential of circulating tumor DNA for the management of patients with lymphoma. JCO Oncol Pract.

[149] Spina V, Bruscaggin A, Cuccaro A, Martini M, Di Trani M, Forestieri G (2018). Circulating tumor DNA reveals genetics, clonal evolution, and residual disease in classical Hodgkin lymphoma. Blood.

[150] Kurtz DM, Scherer F, Jin MC, Soo J, Craig AFM, Esfahani MS (2018). Circulating tumor DNA measurements as early outcome predictors in diffuse large B-cell lymphoma. J Clin Oncol.

[151] Scherer F, Kurtz DM, Newman AM, Stehr H, Craig AFM, Esfahani MS (2016). Distinct biological subtypes and patterns of genome evolution in lymphoma revealed by circulating tumor DNA. Sci Transl Med.

[152] Bruscaggin A, di Bergamo LT, Spina V, Hodkinson B, Forestieri G, Bonfiglio F (2021). Circulating tumor DNA for comprehensive noninvasive monitoring of lymphoma treated with ibrutinib plus nivolumab. Blood Adv.

[153] Roschewski M, Dunleavy K, Pittaluga S, Moorhead M, Pepin F, Kong K (2015). Circulating tumour DNA and CT monitoring in patients with untreated diffuse large B-cell lymphoma: a correlative biomarker study. Lancet Oncol.

[154] Miljkovic MD, Melani C, Pittaluga S, Lakhotia R, Lucas N, Jacob A (2021). Next-generation sequencing-based monitoring of circulating tumor DNA reveals clonotypic heterogeneity in untreated PTCL. Blood Adv.

[155] Kim SJ, Kim YJ, Yoon SE, Ryu KJ, Park B, Park D (2023). Circulating tumor DNA-based genotyping and monitoring for predicting disease relapses of patients with peripheral T-cell lymphomas. Cancer Res Treat.

[156] Rossi D, Diop F, Spaccarotella E, Monti S, Zanni M, Rasi S (2017). Diffuse large B-cell lymphoma genotyping on the liquid biopsy. Blood.

[157] Fernández-Miranda I, Pedrosa L, Llanos M, Franco FF, Gómez S, Martín-Acosta P (2023). Monitoring of circulating tumor DNA predicts response to treatment and early progression in follicular lymphoma: results of a prospective pilot study. Clin Cancer Res.

[158] Jiménez-Ubieto A, Poza M, Martin-Muñoz A, Ruiz-Heredia Y, Dorado S, Figaredo G (2023). Real-life disease monitoring in follicular lymphoma patients using liquid biopsy ultra-deep sequencing and PET/CT. Leukemia.

[159] Kurtz DM, Soo J, Co Ting Keh L, Alig S, Chabon JJ, Sworder BJ (2021). Enhanced detection of minimal residual disease by targeted sequencing of phased variants in circulating tumor DNA. Nat Biotechnol.

[160] Meriranta L, Alkodsi A, Pasanen A, Lepistö M, Mapar P, Blaker YN (2022). Molecular features encoded in the ctDNA reveal heterogeneity and predict outcome in high-risk aggressive B-cell lymphoma. Blood.

[161] Bourbon E, Alcazer V, Cheli E, Huet S, Sujobert P (2021). How to obtain a high quality ctDNA in lymphoma patients: preanalytical tips and tricks. Pharmaceuticals.

[162] Roschewski M, Rossi D, Kurtz DM, Alizadeh AA, Wilson WH (2022). Circulating tumor DNA in lymphoma: principles and future directions. Blood Cancer Discov.

